# Interactome analysis of the lymphocytic choriomeningitis virus nucleoprotein in infected cells reveals ATPase Na^+^/K^+^ transporting subunit Alpha 1 and prohibitin as host-cell factors involved in the life cycle of mammarenaviruses

**DOI:** 10.1371/journal.ppat.1006892

**Published:** 2018-02-20

**Authors:** Masaharu Iwasaki, Petra Minder, Yíngyún Caì, Jens H. Kuhn, John R. Yates, Bruce E. Torbett, Juan C. de la Torre

**Affiliations:** 1 Department of Immunology and Microbiology, The Scripps Research Institute, La Jolla, California, United States of America; 2 Integrated Research Facility at Fort Detrick, Division of Clinical Research, National Institute of Allergy and Infectious Diseases, National Institutes of Health, Frederick, Maryland, United States of America; 3 Department of Molecular Medicine, The Scripps Research Institute, La Jolla, California, United States of America; Harvard Medical School, UNITED STATES

## Abstract

Several mammalian arenaviruses (mammarenaviruses) cause hemorrhagic fevers in humans and pose serious public health concerns in their endemic regions. Additionally, mounting evidence indicates that the worldwide-distributed, prototypic mammarenavirus, lymphocytic choriomeningitis virus (LCMV), is a neglected human pathogen of clinical significance. Concerns about human-pathogenic mammarenaviruses are exacerbated by of the lack of licensed vaccines, and current anti-mammarenavirus therapy is limited to off-label use of ribavirin that is only partially effective. Detailed understanding of virus/host-cell interactions may facilitate the development of novel anti-mammarenavirus strategies by targeting components of the host-cell machinery that are required for efficient virus multiplication. Here we document the generation of a recombinant LCMV encoding a nucleoprotein (NP) containing an affinity tag (rLCMV/Strep-NP) and its use to capture the NP-interactome in infected cells. Our proteomic approach combined with genetics and pharmacological validation assays identified ATPase Na^+^/K^+^ transporting subunit alpha 1 (ATP1A1) and prohibitin (PHB) as pro-viral factors. Cell-based assays revealed that ATP1A1 and PHB are involved in different steps of the virus life cycle. Accordingly, we observed a synergistic inhibitory effect on LCMV multiplication with a combination of ATP1A1 and PHB inhibitors. We show that ATP1A1 inhibitors suppress multiplication of Lassa virus and Candid#1, a live-attenuated vaccine strain of Junín virus, suggesting that the requirement of ATP1A1 in virus multiplication is conserved among genetically distantly related mammarenaviruses. Our findings suggest that clinically approved inhibitors of ATP1A1, like digoxin, could be repurposed to treat infections by mammarenaviruses pathogenic for humans.

## Author summary

Viral hemorrhagic fever-causing mammalian viruses of the family *Arenaviridae* pose serious threats to humans in Africa and South America as the associated infections are highly lethal. The worldwide-distributed lymphocytic choriomeningitis virus (LCMV) is a relative of these dangerous viruses that can be worked with more safely in the laboratory. Although LCMV does not cause viral hemorrhagic fever, it can cause disease in humans. Currently, anti-arenavirus therapy options are very limited, not very effective, and associated with side effects. Development of new therapies has been hampered because knowledge on how arenaviruses interact with proteins of the host cells they infect is limited. Using a modified LCMV, we identified two host-cell proteins called ATPase Na+/K+ transporting subunit alpha 1 (ATP1A1) and prohibitin (PHB) as factors that promote arenavirus infection. Inhibitors of ATP1A1 (cardiac glycosides already used clinically for treatment of other diseases) suppressed multiplication in cell culture of Lassa virus and Junín virus, the two most significant viral hemorrhagic fever-causing mammarenaviruses. Therefore, our data suggest that these inhibitors could be used clinically to treat people infected with arenaviruses.

## Introduction

Mammarenaviruses (*Arenaviridae*: *Mammarenavirus*) cause chronic infections of rodents worldwide [1]. Invasion of human dwellings by infected rodents can result in human infections through mucosal exposure to aerosols or by direct contact of abraded skin with infectious material. Several mammarenaviruses cause viral hemorrhagic fevers (VHFs) in humans and pose important public health problems in their endemic areas [2–6]. Mammarenaviruses are classified into two main groups, Old World (OW) and New World (NW) [1]. The OW Lassa virus (LASV), causative agent of Lassa fever (LF), is the most significant OW mammarenaviral pathogen. LASV is estimated to infect several hundred thousand individuals annually in Western Africa, resulting in a high number of LF cases associated with high morbidity and lethality. Moreover, LASV endemic regions are expanding [7], and the association of the recently identified mammarenavirus Lujo virus with a VHF outbreak in Southern Africa [8, 9] has raised concerns about the emergence of novel VHF-causing mammarenaviruses. The most significant NW mammarenavirus is Junín virus (JUNV), which causes Argentinian hemorrhagic fever [10]. The worldwide-distributed OW mammarenavirus lymphocytic choriomeningitis virus (LCMV) is a neglected human pathogen of clinical significance especially in congenital infections [11–15]. Moreover, LCMV poses a particular threat to immunocompromised individuals, as has been illustrated by fatal cases of LCMV infection associated with organ transplants [16, 17]. However, LCMV research can be safely performed at BSL-2 containment, rather than the BSL-4 containment necessary for live LASV or JUNV research [18].

No US Food and Drug Administration (FDA)-licensed vaccines are available for the treatment of arenavirus infections, although a live attenuated vaccine strain of JUNV, Candid#1, is licensed in Argentina. Likewise, current anti-mammarenavirus therapy is limited to an off-label use of the nucleoside analogue ribavirin that is only partially effective and can cause significant side effects [19–21]. Development of effective anti-mammarenavirus drugs has been hampered by the lack of detailed understanding of virus/host-cell interactions required for mammarenavirus multiplication that could represent amenable targets for antiviral therapy. Such therapies include repurposing of already existing, FDA-approved drugs with strong safety profiles.

Mammarenaviruses produce enveloped virions containing bi-segmented ambisense RNA genomes [1]. Each genome segment, large (L) and small S (small), directs the synthesis of two proteins from open readings frames that are separated by a non-coding intergenic region (IGR) [1]. The S RNA segment encodes the viral nucleoprotein (NP) and glycoprotein precursor (GPC). GPC is co-translationally cleaved by signal peptidase to generate a stable signal peptide (SSP) and is post-translationally processed by site 1 protease (S1P) to generate the mature virion surface glycoproteins GP_1_ and GP_2_. These mature glycoproteins together with SSP form the GP complex that mediates virion receptor recognition and cell entry. The L RNA segment encodes the viral RNA-dependent RNA polymerase (L) and the matrix really interesting new gene (RING) finger protein Z, which is bona fide matrix protein analogous to those found in many negative-sense RNA viruses [22, 23].

Mammarenavirus NP is a multifunctional protein that is involved in different steps of the virus life cycle. A primary role of NP is to encapsidate the viral genomic and antigenomic RNAs to form the nucleocapsid templates. These templates together with L form the virus ribonucleoprotein (RNP) complex that is responsible for directing the biosynthetic processes of virus RNA replication and gene transcription. In addition, the C-terminal region of NP contains distinct functional domains involved in interaction with Z, which regulates viral RNA synthesis and may facilitate virion budding [24]. NP also counteracts the host-cell type I interferon (IFN-I) response to infection by blocking the activation of interferon regulatory factor 3 (IRF3) through the retinoic acid inducible gene-I (RIG-I) and mitochondrial antiviral signaling (MAVS) pathway [25, 26]. NP's IFN-I antagonistic activity has been associated with the 3'-5' exoribonuclease functional domain of the DEDDh family located within the NP carboxyl-terminus [25, 27–31]. In addition, amino acid residues located outside the enzyme active site critically contribute to the anti-INF-I activity of the NP of the New World mammarenavirus Tacaribe (TCRV) [32]

Although NP plays central roles in mammarenavirus multiplication and counteracting the anti-viral host defense, there is limited information about how the interaction of NP with host cell proteins may contribute to NP activities. To fill this knowledge gap and identify potential novel host-cell targets that could be exploited for therapeutic purposes, we generated a recombinant LCMV encoding a Strep-tagged NP (rLCMV/Strep-NP) to capture the NP-interactome in LCMV-infected cells. Our proteomics approach combined with genetics and pharmacological studies identified ATPase Na^+^/K^+^ transporting subunit alpha 1 (ATP1A1) and prohibitin (PHB) as pro-viral factors. Use of RNA interference (RNAi)-mediated knock-down and pharmacological inhibitors of ATP1A1 and PHB reduced virus multiplication significantly. Cell-based assays revealed that inhibitors of ATP1A1 and PHB targeted different steps of the virus life cycle. Accordingly, we observed a synergistic inhibitory effect on LCMV multiplication using inhibitors to ATP1A1 and PHB. Importantly, ATP1A1 inhibitors (cardiac glycosides), which are currently being used in the clinic, suppressed multiplication of LASV and JUNV, suggesting that the requirement of ATP1A1 for LCMV multiplication is conserved among genetically distantly related mammarenaviruses. Thus, repurposing cardiac glycosides to treat infections by human pathogenic arenaviruses may be possible.

## Results

### Generation and characterization of rLCMV/Strep-NP and r3LCMV/eGFP-Strep

**Fig 1:**
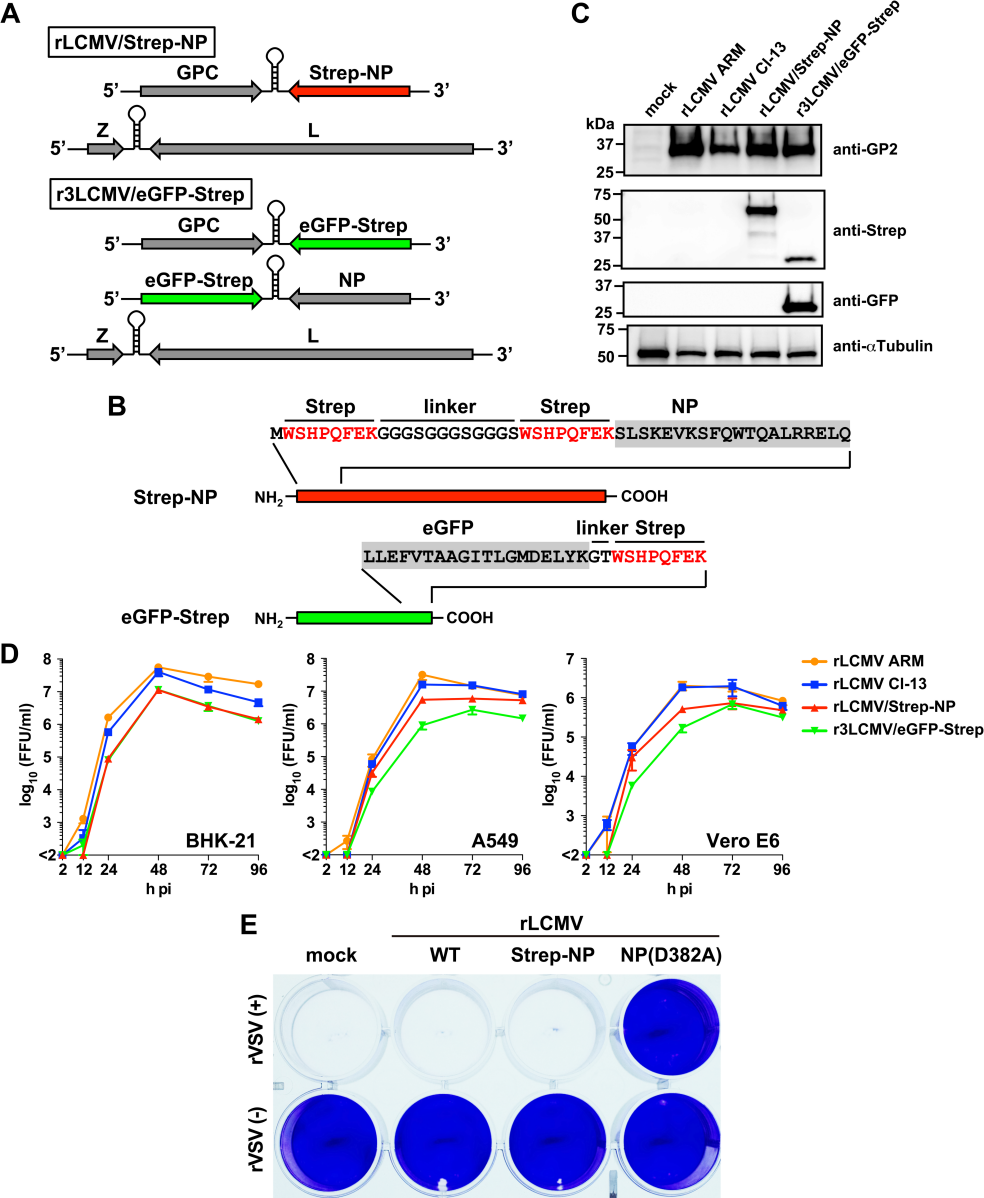
Generation and characterization of rLCMV/Strep-NP and r3LCMV/eGFP-Strep. **(A, B)** Schematic of the genome organization of rLCMVs expressing Strep-NP or eGFP-Strep **(A)** and amino acid composition of N- and C-terminus regions of Strep-NP and eGFP-strep, respectively **(B)**. **(C)** Expression of Strep-tagged proteins. A549 cells seeded (5.0 x 10^5^ cells/well) in a 6-well plate and cultured overnight were infected (MOI = 0.1) with the indicated rLCMVs. At 48 h pi, total cell lysates were prepared, and protein expression was analyzed by western blotting. **(D)** Growth properties of rLCMV expressing Strep-tagged proteins. BHK-21 (1.75 x 10^5^ cells/well), A549 (1.25 x 10^5^ cells/well), or Vero E6 (1.25 x 10^5^ cells/well) cells seeded in 24-well plates and cultured overnight were infected (MOI = 0.01) with the indicated rLCMVs. At the indicated times pi, TCSs were collected and virus titers determined by IFFA. Results represent means ± SD of the results of three independent experiments. **(E)** Lack of induction of IFN-I in cells infected with rLCMV/Strep-NP. A549 cells were infected (MOI = 0.1) with the indicated rLCMV or mock-infected, and 36 h later infected with SeV (MOI = 1). At 24 h pi with SeV, TCS were collected and used, following virus inactivation by U.V., to treat Vero E6 cells for 16 h, followed by infection with rVSV (MOI = 0.1) [rVSV(+)] or mock-infection [rVSV(-)]. At 24 h pi with rVSV, cells were fixed with 4% PFA and stained with crystal violet to assess rVSV-induced cytopathic effect. We used as control rLCMV/NP(D382A) that contains mutation D382A within NP, which has been shown to abrogate the NP’s ability to counteract the induction of IFN-I production.

Plasmid-mediated expression of viral genes in transfected cells has been often used successfully to identify virus-host-cell protein-protein interactions (VH PPI). However, this approach has the potential problem that overexpression of a single viral gene product may potentiate PPI interactions that are not relevant during the course of a natural virus infection. To overcome this issue, we designed a recombinant LCMV (rLCMV) encoding a tandem [WSHPQFEK(GGGS)_3_WSHPQFEK] Strep-tag fused to the amino-terminus of NP (rLCMV/Strep-NP) (**Fig 1A and 1B**). To facilitate the identification of specific PPI between NP and host cell proteins, we used our mammarenavirus tri-segmented (r3) platform [30] to design an r3LCMV expressing a C-terminus Strep-tag version of enhanced green fluorescent protein (r3LCMV/eGFP-Strep) that we used as a negative control (**Fig 1A and 1B**). We rescued rLCMV/Strep-NP and r3LCMV/eGFP-Strep and confirmed the expression of strep-tagged NP and eGFP in rLCMV/Strep-NP- and r3LCMV/eGFP-Strep-infected cells, respectively (**Fig 1C**). Next, we examined the growth properties of rLCMV/Strep-NP and r3LCMV/eGFP-Strep in three different cells lines from hamsters, humans, and nonhuman primates (BHK-21, A549, and Vero E6 cells, respectively) (**Fig 1D**). The fitness of rLCMV/Strep-NP and r3LCMV/eGFP-Strep was modestly decreased compared to that observed with wild-type (WT) Armstrong (rLCMV ARM) and Clone 13 (rLCMV Cl-13) strains of LCMV. However, both rLCMV/Strep-NP and r3LCMV/eGFP-Strep had WT-like growth kinetics and reached high titers. As with WT LCMV, infection with rLCMV/Strep-NP prevented production of bioactive IFN-I by cells in response to Sendai virus (SeV) infection as determined using an IFN bioassay based on protection against the cytopathic effect (CPE) induced by infection with vesicular stomatitis virus (VSV) (**Fig 1E**). Vero cells treated for 16 h with tissue cultured supernatants (TCS) from A549 cells infected first with WT LCMV or rLCMV/Strep, followed by 24 h infection with SeV, remained fully susceptible to VSV- induced CPE. In contrast, Vero cells treated with TCS from A549 cells infected with rLCMV/NP(D382A), a mutant unable to prevent induction of IFN-I [30], and subsequently with SeV, were protected against VSV induced CPE.

### Identification of NP-interacting host-cell proteins in LCMV-infected cells

**Fig 2:**
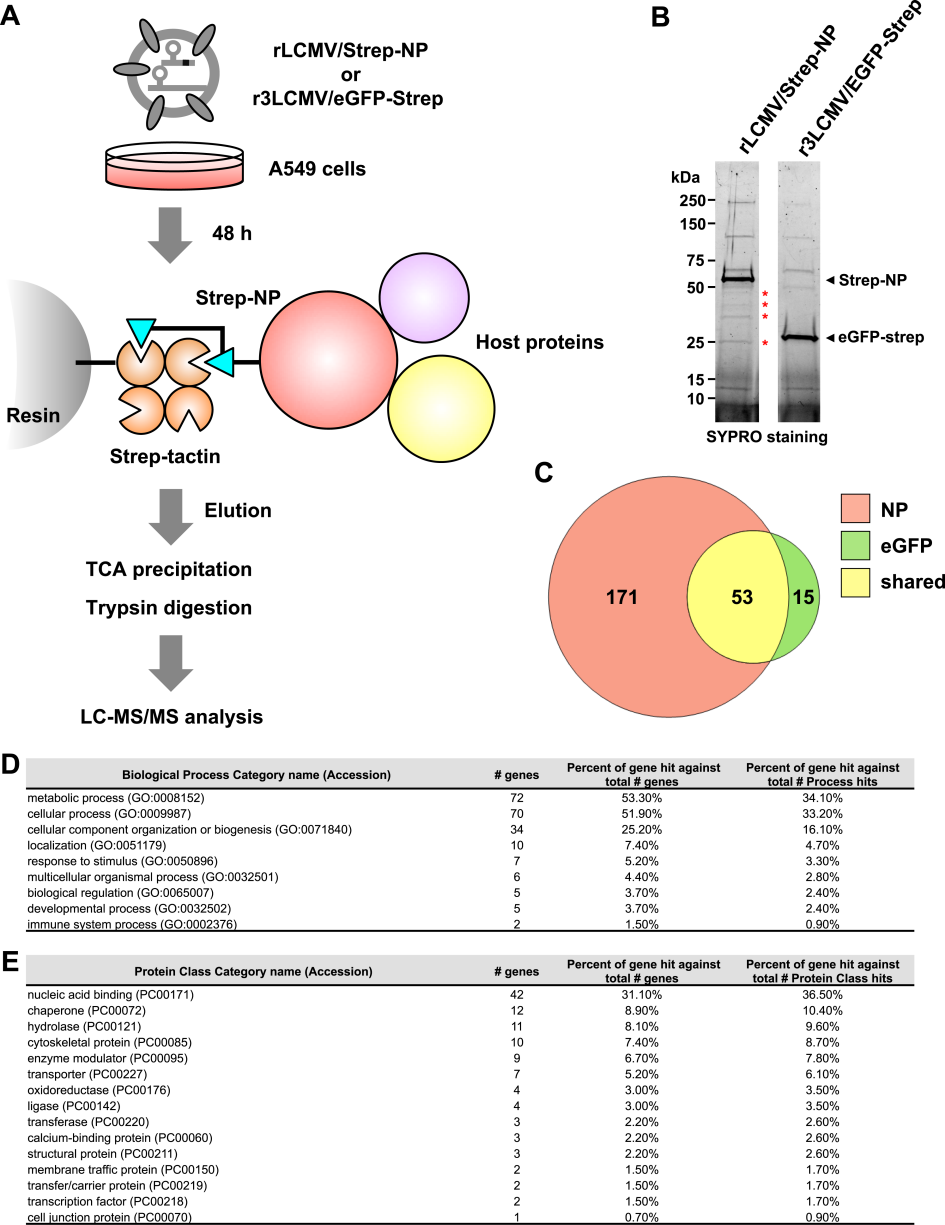
LC-MS/MS analysis of NP-binding proteins. **(A)** Flow chart of the experimental approach to identify NP-interacting host-cell proteins in LCMV-infected cells. A549 cells prepared in six 15-cm dishes (total of 1.0 x 10^8^ cells) were infected (MOI = 0.1) with either rLCMV/Strep-NP or r3LCMV/eGFP. At 48 h pi, total cell lysates were prepared, and NP- or eGFP-interacting proteins were pulled down (PD) using Streptactin-coated sepharose resin. Protein complexes bound to the resin were eluted using 2.5 mM of desthiobiotin. Eluates were precipitated using TCA followed by trypsin digestion. Tryptic peptides were subjected to LC-MS/MS analysis. **(B)** Detection of proteins present in PD samples. Protein complexes present in PD samples were separated by SDS-PAGE and visualized by SYPRO staining. Some protein bands present only in the Strep-NP PD sample are indicated by asterisks. **(C)** Venn diagram of the NP- and eGFP-interacting proteins identified by LC-MS/MS analysis. **(D, E)** Gene Onthology (GO) analysis of the NP-interacting proteins identified by LC-MS/MS. Bioinformatic analysis by PANTHER was performed showing the number of genes of identified NP-interacting proteins classified by biological process **(D)** and protein class **(E)**.

We selected the human A549 cell line because lung epithelial cells are one of the initial cell targets of humans following inhalation of mammarenavirions. We infected A549 cells (multiplicity of infection [MOI] = 0.1) with either rLCMV/Strep-NP or r3LCMV/eGFP-Strep (**Fig 2A**). At 48 h post-inoculation (pi), we prepared total cell lysates for pull-down (PD) assays using a sepharose resin coated with strep-tactin. Aliquots of the protein complexes present in the PD samples were fractionated by sodium dodecyl sulfate-polyacrylamide gel electrophoresis (SDS-PAGE) (**Fig 2B**) followed by SYPRO Ruby protein gel staining. We compared the pattern of stained protein bands detected between rLCMV/Strep-NP- and r3LCMV/eGFP-Strep-infected samples and confirmed the presence of Strep-NP and eGFP-Strep in pertinent samples (**Fig 2B**). Protein complexes in the rest of eluates from the PD samples were concentrated by trichloroacetic acid (TCA) precipitation and subjected to trypsin digestion (**Fig 2A**). Digested peptides were subjected to liquid chromatography-tandem mass spectrometry (LC-MS/MS) analysis using a hybrid mass spectrometer consisting of linear quadrupole ion dual cell trap (LTQ) Velos and an Orbitrap analyser.

We classified host-cell proteins identified by LC-MS/MS analysis from two independent biological replicates into two groups: 1) proteins found only in Strep-NP PD samples with at least five spectral counts (**Table 1**), and 2) proteins found in both Strep-NP and eGFP-Strep PD samples with five or higher spectral counts in Strep-NP samples and at least 2-fold higher spectral counts in Strep-NP PD compared to eGFP PD samples (**Table 2**). Filtering the data using these criteria resulted in identification of 139 candidate host-cell proteins as NP-interacting partners. Among 53 proteins found present in both NP- and eGFP-PD samples, 36 had spectral counts in the NP-PD sample that were less than two-fold higher than their corresponding spectral counts in the eGFP-PD sample (**Fig 2C and S1 Table**). Based on the criteria we described above, we considered these proteins to be highly unlikely specific NP-interactors with an involvement in the LCMV life cycle, and therefore we did not consider these hits for further analysis. However, we acknowledge that we cannot formally rule out that some of these hits could still play a role on the life cycle of LCMV.

**Table 1:** List of identified host-cell proteins found only in NP-strep pull down samples with at least five spectral counts.

Description	Gene Symbol	Accession	Spectral count		
			Exp. 1	Exp. 2	Ave.
T-complex protein 1 subunit theta	CCT8	P50990	467	320	393.5
T-complex protein 1 subunit delta	CCT4	P50991	394	80	237
T-complex protein 1 subunit gamma	CCT3	P49368	343	123	233
T-complex protein 1 subunit eta	CCT7	Q99832	354	76	215
T-complex protein 1 subunit alpha	TCP1	P17987	241	116	178.5
Tubulin alpha-4A chain	TUBA4A	P68366	144	23	83.5
T-complex protein 1 subunit zeta	CCT6A	P40227	102	64	83
T-complex protein 1 subunit beta	CCT2	P78371	94	60	77
T-complex protein 1 subunit epsilon	CCT5	P48643	97	42	69.5
Tubulin beta-2A chain	TUBB2A	Q13885	96	21	58.5
Alpha-actinin-4	ACTN4	O43707	82	18	50
Filamin-B	FLNB	O75369	65	31	48
Nuclear cap-binding protein subunit 1	NCBP1	Q09161	51	36	43.5
Cytoplasmic FMR1-interacting protein 1	CYFIP1	Q7L576	60	21	40.5
40S ribosomal protein SA	RPSA	P08865	25	41	33
Aldo-keto reductase family 1 member B10	AKR1B10	O60218	59	4	31.5
40S ribosomal protein S3a	RPS3A	P61247	42	12	27
Sarcoplasmic/endoplasmic reticulum calcium ATPase 2	ATP2A2	P16615	21	28	24.5
Cell cycle and apoptosis regulator protein 2	CCAR2	Q8N163	31	17	24
Probable ATP-dependent RNA helicase DDX6	DDX6	P26196	44	2	23
Heterogeneous nuclear ribonucleoprotein H	HNRNPH1	P31943	40	6	23
Polyadenylate-binding protein 1	PABPC1	P11940	39	6	22.5
40S ribosomal protein S17	RPS17	P08708	41	2	21.5
40S ribosomal protein S17-like	RPS17L	P0CW22	41	2	21.5
RNA-binding protein 25	RBM25	P49756	33	8	20.5
Y-box-binding protein 3	YBX3	P16989	36	4	20
40S ribosomal protein S19	RPS19	P39019	26	14	20
4F2 cell-surface antigen heavy chain	SLC3A2	P08195	29	10	19.5
40S ribosomal protein S16	RPS16	P62249	34	4	19
Replication protein A 70 kDa DNA-binding subunit	RPA1	P27694	26	10	18
Prelamin-A/C	LMNA	P02545	25	8	16.5
ADP/ATP translocase 2	SLC25A5	P05141	27	6	16.5
Keratin, type II cytoskeletal 7	KRT7	P08729	19	14	16.5
40S ribosomal protein S4, X isoform	RPS4X	P62701	22	10	16
Ribosome-binding protein 1	RRBP1	Q9P2E9	21	11	16
Nck-associated protein 1	NCKAP1	Q9Y2A7	21	11	16
40S ribosomal protein S2	RPS2	P15880	22	7	14.5
Heat shock protein HSP 90-beta	HSP90AB1	P08238	11	17	14
ADP/ATP translocase 3	SLC25A6	P12236	24	4	14
Alpha-actinin-1	ACTN1	P12814	23	5	14
RNA-binding protein 14	RBM14	Q96PK6	22	6	14
RNA-binding motif protein, X chromosome	RBMX	P38159	24	3	13.5
Prefoldin subunit 2	PFDN2	Q9UHV9	15	12	13.5
Probable ATP-dependent RNA helicase DDX5	DDX5	P17844	6	20	13
Filamin-C	FLNC	Q14315	24	2	13
Probable ATP-dependent RNA helicase DDX17	DDX17	Q92841	6	20	13
Neuroblast differentiation-associated protein AHNAK	AHNAK	Q09666	15	10	12.5
60S ribosomal protein L23	RPL23	P62829	12	12	12
Cytoplasmic FMR1-interacting protein 2	CYFIP2	Q96F07	15	8	11.5
Keratin, type I cytoskeletal 19	KRT19	P08727	5	17	11
Dihydrolipoyl dehydrogenase, mitochondrial	DLD	P09622	19	3	11
ATP synthase subunit alpha, mitochondrial	ATP5A1	P25705	7	15	11
DNA-dependent protein kinase catalytic subunit	PRKDC	P78527	10	12	11
Splicing factor 3B subunit 3	SF3B3	Q15393	8	14	11
RuvB-like 2	RUVBL2	Q9Y230	10	11	10.5
Apoptosis-inducing factor 1, mitochondrial	AIFM1	O95831	3	17	10
Heterogeneous nuclear ribonucleoproteins A2/B1	HNRNPA2B1	P22626	18	2	10
Protein disulfide-isomerase A6	PDIA6	Q15084	8	12	10
Histone H1x	H1FX	Q92522	17	3	10
CAD protein	CAD	P27708	8	11	9.5
Heterogeneous nuclear ribonucleoprotein H3	HNRNPH3	P31942	15	4	9.5
Heat shock protein HSP 90-alpha	HSP90AA1	P07900	8	10	9
Endoplasmin	HSP90B1	P14625	9	9	9
Large neutral amino acids transporter small subunit 1	SLC7A5	Q01650	6	12	9
Keratin, type II cuticular Hb1	KRT81	Q14533	10	8	9
Putative helicase MOV-10	MOV10	Q9HCE1	8	10	9
Microtubule-associated protein 1B	MAP1B	P46821	6	11	8.5
Spectrin beta chain, non-erythrocytic 1	SPTBN1	Q01082	7	10	8.5
60S ribosomal protein L18	RPL18	Q07020	7	10	8.5
Pre-mRNA-processing-splicing factor 8	PRPF8	Q6P2Q9	14	3	8.5
60 kDa SS-A/Ro ribonucleoprotein	TROVE2	P10155	13	3	8
40S ribosomal protein S9	RPS9	P46781	11	5	8
Nuclear cap-binding protein subunit 2	NCBP2	P52298	7	9	8
39S ribosomal protein L12, mitochondrial	MRPL12	P52815	10	6	8
40S ribosomal protein S11	RPS11	P62280	13	3	8
Small nuclear ribonucleoprotein Sm D2	SNRPD2	P62316	3	13	8
Tight junction protein ZO-1	TJP1	Q07157	8	8	8
116 kDa U5 small nuclear ribonucleoprotein component	EFTUD2	Q15029	11	5	8
Sodium/potassium-transporting ATPase subunit alpha-1	ATP1A1	P05023	3	12	7.5
Low molecular weight phosphotyrosine protein phosphatase	ACP1	P24666	6	9	7.5
Myosin-9	MYH9	P35579	13	2	7.5
40S ribosomal protein S25	RPS25	P62851	5	10	7.5
Cytoplasmic dynein 1 heavy chain 1	DYNC1H1	Q14204	7	8	7.5
Eukaryotic translation initiation factor 3 subunit L	EIF3L	Q9Y262	6	9	7.5
Septin-9	SEPT9	Q9UHD8	6	8	7
28S ribosomal protein S18b, mitochondrial	MRPS18B	Q9Y676	5	9	7
Keratin, type II cytoskeletal 75	KRT75	O95678	7	6	6.5
Proteasome subunit beta type-5	PSMB5	P28074	6	7	6.5
Prohibitin	PHB	P35232	6	7	6.5
40S ribosomal protein S6	RPS6	P62753	4	9	6.5
60S ribosomal protein L19	RPL19	P84098	8	5	6.5
2-oxoglutarate dehydrogenase, mitochondrial	OGDH	Q02218	6	7	6.5
Leucine-rich repeat flightless-interacting protein 1	LRRFIP1	Q32MZ4	6	7	6.5
Thioredoxin domain-containing protein 5	TXNDC5	Q8NBS9	4	9	6.5
60S acidic ribosomal protein P0	RPLP0	P05388	5	7	6
U1 small nuclear ribonucleoprotein 70 kDa	SNRNP70	P08621	8	4	6
Fatty acid synthase	FASN	P49327	6	6	6
Poly(rC)-binding protein 1	PCBP1	Q15365	4	8	6
Septin-7	SEPT7	Q16181	5	7	6
Heat shock protein 105 kDa	HSPH1	Q92598	8	4	6
39S ribosomal protein L11, mitochondrial	MRPL11	Q9Y3B7	5	7	6
Wiskott-Aldrich syndrome protein family member 2	WASF2	Q9Y6W5	9	3	6
Lupus La protein	SSB	P05455	6	5	5.5
Bifunctional glutamate/proline—tRNA ligase	EPRS	P07814	9	2	5.5
Histone H1.5	HIST1H1B	P16401	3	8	5.5
Elongation factor 1-delta	EEF1D	P29692	5	6	5.5
Transgelin-2	TAGLN2	P37802	7	4	5.5
Arginine—tRNA ligase, cytoplasmic	RARS	P54136	7	4	5.5
60S ribosomal protein L11	RPL11	P62913	2	9	5.5
Elongation factor 1-alpha 1	EEF1A1	P68104	6	5	5.5
Non-POU domain-containing octamer-binding protein	NONO	Q15233	8	3	5.5
Mitochondrial import inner membrane translocase subunit TIM50	TIMM50	Q3ZCQ8	9	2	5.5
DNL-type zinc finger protein	DNLZ	Q5SXM8	9	2	5.5
Putative elongation factor 1-alpha-like 3	EEF1A1P5	Q5VTE0	6	5	5.5
Heterogeneous nuclear ribonucleoprotein U-like protein 1	HNRNPUL1	Q9BUJ2	8	3	5.5
Myosin-4	MYH4	Q9Y623	2	9	5.5
Heat shock protein beta-1	HSPB1	P04792	6	4	5
60S ribosomal protein L7	RPL7	P18124	5	5	5
60S ribosomal protein L29	RPL29	P47914	3	7	5
Clathrin heavy chain 1	CLTC	Q00610	8	2	5
LIM and SH3 domain protein 1	LASP1	Q14847	8	2	5
UPF0515 protein C19orf66	C19orf66	Q9NUL5	6	4	5

**Table 2:** Identified host-cell proteins with spectral counts at least 2-fold higher in NP pull down samples than eGFP samples.

Description	Gene Symbol	Accession	Spectral count						Fold change
			NP			eGFP			
			Exp. 1	Exp. 2	Ave.	Exp. 1	Exp. 2	Ave.	NP/EGFP
Tubulin alpha-1B chain	TUBA1B	P68363	169	51	110	6	27	16.5	6.666666667
Tubulin alpha-1C chain	TUBA1C	Q9BQE3	166	50	108	6	27	16.5	6.545454545
Nuclear fragile X mental retardation-interacting protein 2	NUFIP2	Q7Z417	358	40	199	21	51	36	5.527777778
Tubulin beta chain	TUBB	P07437	122	26	74	3	39	21	3.523809524
Filamin-A	FLNA	P21333	216	82	149	7	81	44	3.386363636
Guanine nucleotide-binding protein subunit beta-2-like 1	GNB2L1	P63244	80	8	44	3	24	13.5	3.259259259
Tubulin beta-4B chain	TUBB4B	P68371	110	24	67	7	36	21.5	3.11627907
Ataxin-2-like protein	ATXN2L	Q8WWM7	367	191	279	38	155	96.5	2.89119171
40S ribosomal protein S18	RPS18	P62269	76	41	58.5	13	28	20.5	2.853658537
Heat shock cognate 71 kDa protein	HSPA8	P11142	275	89	182	37	109	73	2.493150685
YTH domain-containing family protein 3	YTHDF3	Q7Z739	8	16	12	7	3	5	2.4
Heat shock-related 70 kDa protein 2	HSPA2	P54652	110	67	88.5	32	44	38	2.328947368
Pre-mRNA-processing factor 40 homolog A	PRPF40A	O75400	43	8	25.5	5	17	11	2.318181818
Stress-70 protein, mitochondrial	HSPA9	P38646	369	117	243	61	174	117.5	2.068085106
Protein SET	SET	Q01105	17	16	16.5	11	5	8	2.0625
Nuclease-sensitive element-binding protein 1	YBX1	P67809	54	11	32.5	28	4	16	2.03125
Transitional endoplasmic reticulum ATPase	VCP	P55072	31	11	21	4	17	10.5	2

The protein annotation through evolutionary relationship (PANTHER) protein family classification (Biological Process) of the NP-interacting protein candidates revealed that a large number of proteins were involved in metabolic and cellular processes (**Fig 2D**). We also analyzed the molecular functions of the NP-interacting protein candidates according to the PANTHER protein profile classification (**Fig 2E**), which revealed diversified biochemical functions enriched for nucleic acid-binding proteins and chaperones.

### Effect of RNAi-mediated knock down expression of NP-interacting host-cell proteins on LCMV multiplication

**Fig 3:**
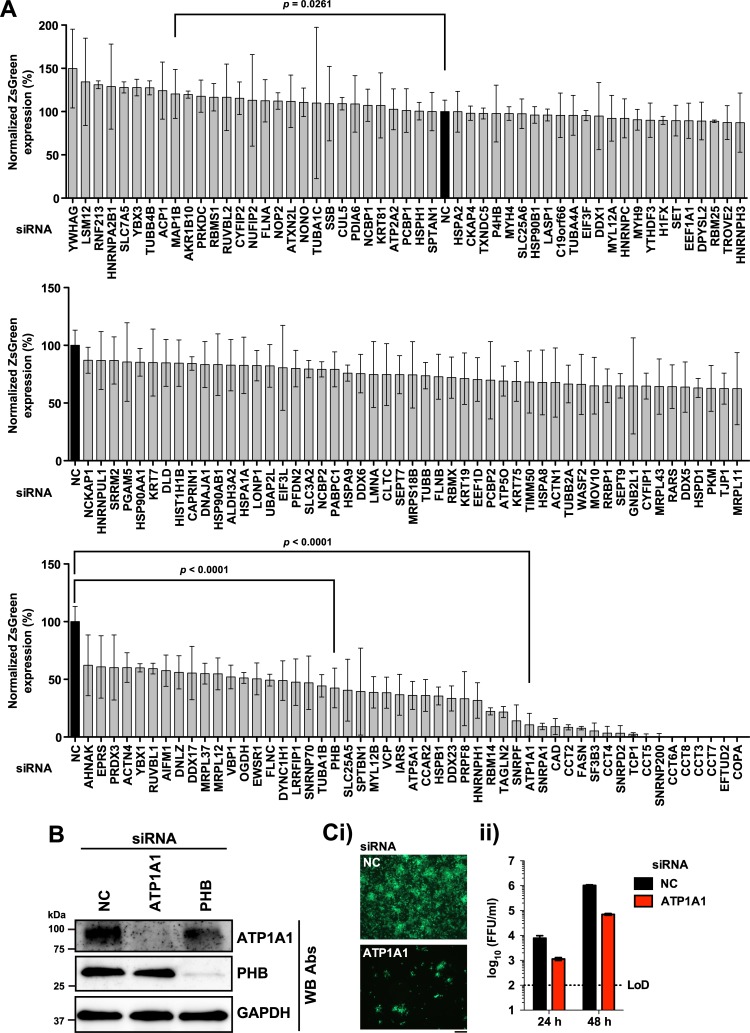
Effect of knock-down of genes identified LC-MS/MS analysis on LCMV multiplication. **(A)** Effect of siRNA-mediated knockdown expression of genes identified by proteomics analysis. A549 cells (1,000 cells/well) in a 384-well plate were reverse transfected with siRNA pools targeting each indicated gene. At 72 h post-transfection, cells were infected (MOI = 0.05) with rLCMV/ZsG. At 48 h pi, cells were fixed and stained with DAPI. ZsGreen and DAPI signals were measured by a fluorescence plate reader. ZsGreen signal was normalized to DAPI signal. Normalized values from cells transfected with the control (NC, black bar) non-specific siRNA was set to 100%. A panel of 154 siRNAs including NC siRNA was evaluated three independent times. Results from three independent experiments were sorted by mean values and assigned to three graphs with SD. Each graph includes identical NC results (black bar) for reference. **(B)** Reduction of protein expression of ATP1A1 and PHB by siRNA-mediated gene knockdown. A549 cells (3.0 x 10^4^ cells/well) were reverse transfected in a 24-well plate with siRNA pools against either ATP1A1 or PHB or with NC siRNA. At 72 h post-transfection, total cell lysate was prepared, and expression of ATP1A1 and PHB in cell lysates were determined by western blots. **(C)** Effect of siRNA-mediated kd of ATP1A1 on production of infectious LCMV progeny. A549 cells (1.5 x 10^4^ cells/well; 48-well plate) were reverse-transfected with siRNAs against ATP1A1 or with NC siRNA. 72 h later, cells were infected (MOI = 0.01) with rLCMV/ZsG. At 24 h and 48 h pi, TCSs were collected. At 48 h pi, cells were fixed and ZsGreen expression examined by fluorescence microscopy (i). Bar, 200 μm. Virus titers in TCSs were determined by IFFA (ii). Data represent means ± SD of results from three independent experiments. LoD, limit of detection.

To initially assess pro- or anti-viral roles of NP-interacting host-cell proteins identified by LC-MS/MS, we examined the effect of small interfering RNA (siRNA)-mediated knockdown (kd) of each of the corresponding genes on multiplication of rLCMV expressing reporter gene ZsGreen (ZsG) in A549 cells (**Fig 3A**). The siRNAs we used were from the genome-wide ON TARGET-Plus (OTP) Human siRNA library (18,301 genes, 4 siRNAs/gene). OTPs are latest generation of siRNAs and offer significant advantages over previous generations. Off-target effects are primarily driven by antisense strand microRNA (miR)-like seed activity. In OTPs, the sense strand is modified to favor antisense strand uptake whereas the antisense strand seed region is modified to drastically reduce seed-related off-targeting [33]. In addition, OTPs are designed on the foundation of the SMARTselection algorithm (Dharmacon), widely considered to be the best algorithm for rational siRNA design strategy. Numerous host-cell factors showed an anti-LCMV effect (increased ZsG expression by kd of the genes), including microtubule-associated protein 1B (MAP1B) [34], which has been shown to inhibit multiplication of other viruses. Host-cell factors exhibiting pro-viral activity (decreased ZsG expression by kd of the gene) included ATP1A1 and PHB, which are involved in multiplication of a variety of viruses including Ebola virus [35], coronaviruses [36], hepatitis C virus (HCV) [37], influenza A virus (FLUAV) subtype H5N1 [38], dengue virus 2 (DENV-2) [39], and chikungunya virus (CHIKV) [40].

**Fig 4:**
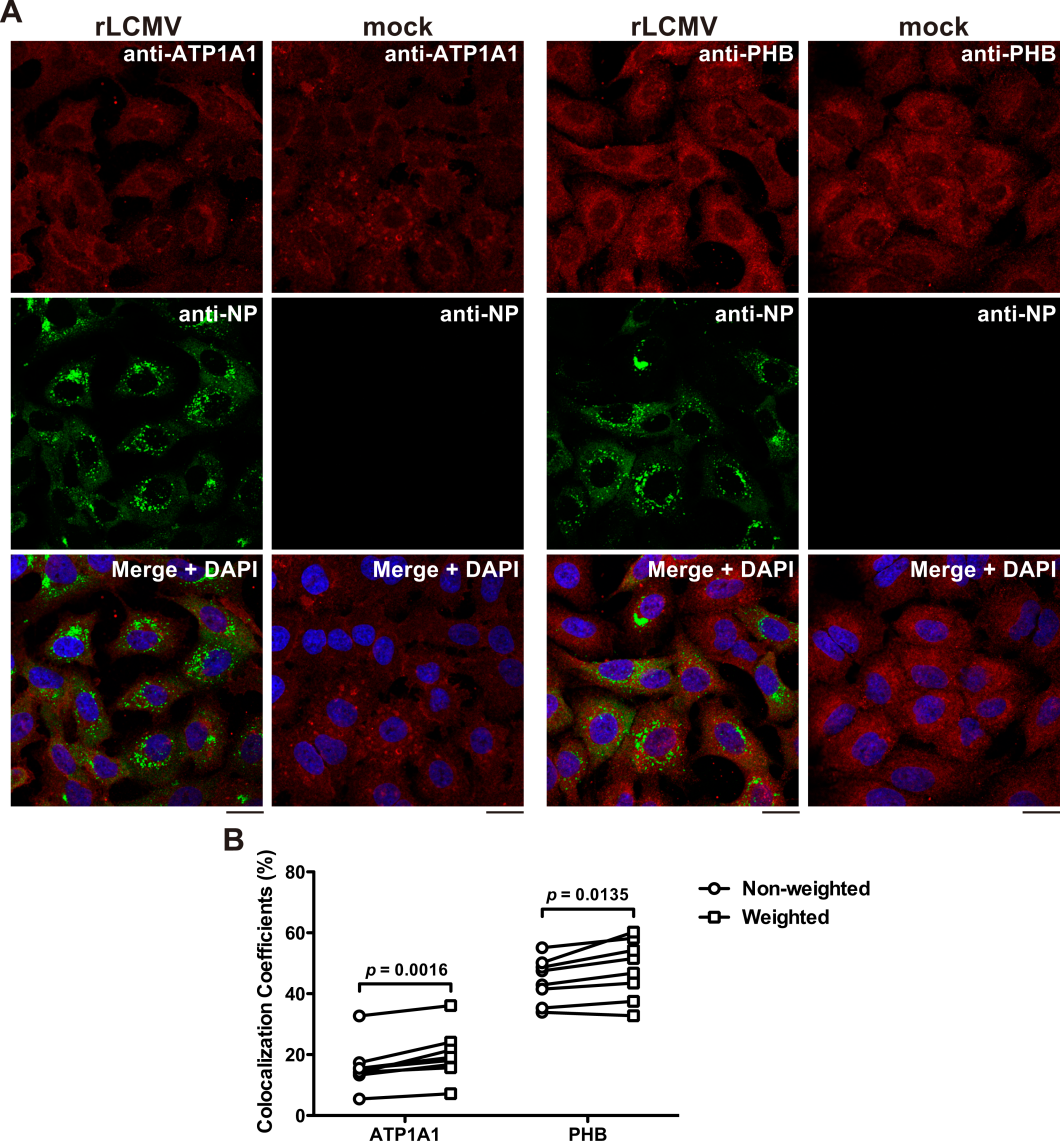
Co-localization of NP with ATP1A1 and PHB. **(A)** Intracellular distributions of NP and ATP1A1 or PHB. A549 cells seeded (5 x 10^4^ cells/well) in a 24-well plate and cultured overnight were infected (MOI = 0.1) with rLCMV Cl-13 or remained uninfected (mock). At 48 h pi, cells were fixed, stained with primary mouse anti ATP1A1 or PHB antibody followed by secondary anti-mouse IgG antibody conjugated with Alexa Fluor 568 (anti-mouse IgG-AF568). Subsequently, cells were stained with VL-4-AF488 (anti-NP), and observed by a confocal microscope. Bars, 20 μm. **(B)** Comparison of non-weighted and weighted co-localization coefficients (CC). Non-weighted CC were determined by dividing the sum of both green and red positive pixels by the sum of green-positive pixels. Thresholds were determined based on the signal intensity of mock-infected sample stained with VL-4-AF488 and anti-mouse IgG-AF568. Weighted CC were determined by taking into consideration the brightness of each channel signal. *p* values were determined by a two-tailed paired *t* test using GraphPad Prism software.

To begin assessing the potential biological implications of the identified NP-host cell protein interactions, we selected ATP1A1 and PHB given the availability of reagents, existing knowledge about their roles in cell physiology, and evidence of participation in multiplication of other viruses. We confirmed that cells transfected with siRNA specific to ATP1A1 and PHB exhibited the predicted reduced levels in ATP1A1 and PHB protein expression (**Fig 3B**). Likewise, we examined whether siRNA-mediated reduced expression levels of ZsGreen correlated with reduced LCMV progeny titers. For this, we transfected A549 cells with siRNA targeting ATP1A1 or with NC siRNA 72 h prior to infection with rLCMV/ZsG. We found that siRNA-mediated kd of ATP1A1 dramatically inhibited ZsGreen expression (**Fig 3Ci**), which was associated with a significant reduction of infectious LCMV progeny (**Fig 3Cii**). Our attempts to see interaction between NP and ATP1A1 or NP and PHB by co-immunoprecipitation were unsuccessful. Several possibilities could account for this, including interactions of low affinity or high on/off rate or both. Another consideration is that only a minor fraction of NP might be engaged in the interaction with a given host cell protein, and therefore, detection of these interactions would require highly sensitive methods such as LC-MS/MS. To overcome this problem we used confocal microscopy to examine the co-localization of NP with ATP1A1 and PHB in LCMV-infected cells. Weighted co-localization coefficients (CC), determined by taking into consideration the brightness of each channel signal, were significantly higher than non-weighted CC, indicating the presence of brighter pixels in the co-localized regions compared to the non-co-localized regions (**Fig 4**).

### Effect of pharmacological inhibition of ATP1A1 and PHB on LCMV multiplication

The cardiac glycoside ouabain is an inhibitor of ATP1A1 that has been used to treat congestive heart failure in European countries [41]. The PHB inhibitor rocaglamide is a flavagline from an *Aglaia* tree used in traditional Chinese medicine [42] that has potent anticancer activity [43]. To examine whether pharmacological inhibition of ATP1A1 or PHB inhibited LCMV multiplication, we pretreated human (A549 and HEK 293T), nonhuman primate (Vero E6), and rodent (murine L929 and hamster BHK-21) cells with ouabain or rocaglamide and infected them with rLCMV/eGFP (**S1 Fig**). Ouabain treatment resulted in a strong dose-dependent inhibition of eGFP expression in infected human- and nonhuman primate cells, but did not affect eGFP expression intensity in infected rodent cells (**S1A Fig**). This finding is consistent with rodents expressing an ATP1A1 allele that is resistant to ouabain inhibition [44].

**Fig 5:**
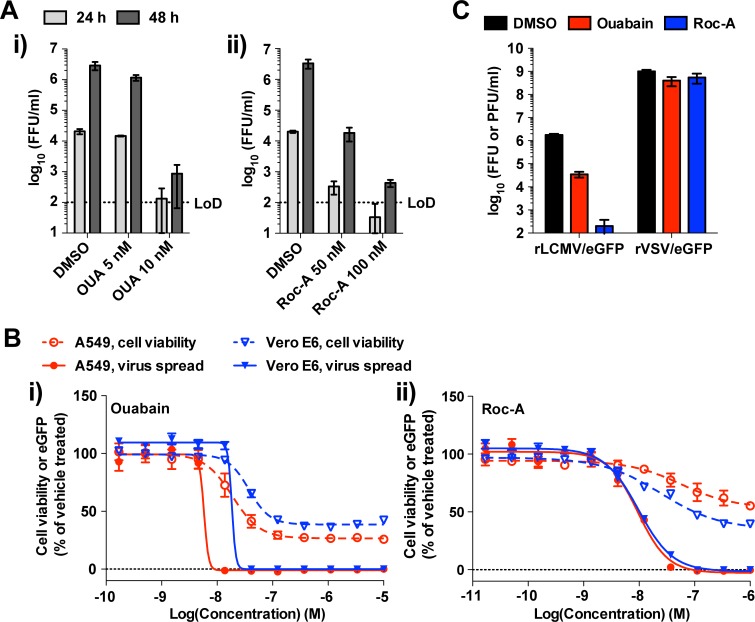
Effect of pharmacological inhibition of ATP1A1 and PHB on LCMV multiplication. **(A)** A549 cells seeded (1.25 x 10^5^ cells/well) in 24-well plates and cultured overnight were treated with either ouabain (OUA) (i) or rocaglamide (Roc-A) (ii) at indicated concentrations or with DMSO (vehicle control) for 2 h and then infected (MOI = 0.01) with rLCMV/eGFP. Compounds were present throughout the experiment. At 24 and 48 h pi, TCSs were collected, and virus titers determined by IFFA. Data represent means ± SD of results from three independent experiments. LoD, the limit of detection. **(B)** Inhibitory effects of ouabain and Roc-A on virus propagation and cell viability. A549 cells seeded (2.0 x 10^4^ cells/well) in 96-well plates and cultured overnight were treated with 3-fold serial dilutions of either ouabain (i) or Roc-A (ii) for 2 h and then infected (MOI = 0.01) with rLCMV/eGFP. Compounds were present throughout the experiment. At 48 h pi, cells were fixed to examine eGFP expression and cell viability as determined by CellTiter 96 AQ_ueous_ one solution reagent. The data represent means ± SD of the results from four (cell viability assay) or six (virus spread assay) replicates. The therapeutic index (TI) was calculated by dividing CC_50_ by IC_50_. **(C)** Effect of ouabain or Roc-A treatment on rVSV/eGFP multiplication. A549 cells seeded (1.25 x 10^5^ cells/well) and cultured overnight were treated with either ouabain (10 nM) Roc-A (100 nM), or vehicle control (DMSO) for 2 h and infected (MOI = 0.01) with either rLCMV/eGFP or rVSV/eGFP. At 72 h pi, TCS was collected, and virus titers were determined by IFFA (rLCMV/eGFP, expressed as FFUs) or a plaque assay (rVSV/eGFP, expressed as PFUs). Compounds were present to study endpoint. Results represent means ± SD of the results of three independent experiments.

Likewise, we observed a dose-dependent rocaglamide inhibition of eGFP expression in all cell lines infected with rLCMV/eGFP (**S1B Fig**). Consistent with these findings, production of infectious LCMV progeny was reduced by treatment with either ouabain or rocaglamide (**Fig 5A**) within a concentration range that had minimal impact on cell viability (**Fig 5B**). To examine the correlation between efficacy and cytotoxicity of these compounds, we determined their therapeutic index (TI = CC_50_/IC_50_). Ouabain had TIs of 4.99 (CC_50_ = 28.9 nM, IC_50_ = 5.79 nM [log10−8.237M]) and 3.81 (CC_50_ = 70.0 nM, IC_50_ = 18.4 nM) in A549 and Vero E6 cells, respectively (**Fig 5Bi**); whereas rocaglamide had TIs of >105 (CC_50_ > 1000 nM, IC_50_ = 9.51 nM) and 10.3 (CC_50_ = 100 nM, IC_50_ = 9.75 nM) in A549 and Vero E6 cells, respectively (**Fig 5Bii**). Moreover, the ATP1A1 antagonist inhibitor, bufalin, also exhibited robust anti-LCMV activity with TIs of 8.92 (CC_50_ = 16.4 nM, IC_50_ = 1.85 nM) and 5.90 (CC_50_ = 73.8 nM, IC_50_ = 12.5 nM) in A549 and Vero E6 cells, respectively (**S2 Fig**). Multiplication of vesicular stomatitis Indiana virus (VSV) was not significantly affected by either ouabain (10 nM) or rocaglamide (100 nM) (**Fig 5C**), further supporting a specific anti-LCMV activity of ouabain and rocaglamide that was not due to reduced cell viability.

### Roles of ATP1A1 and PHB in different steps of the LCMV life cycle

**Fig 6:**
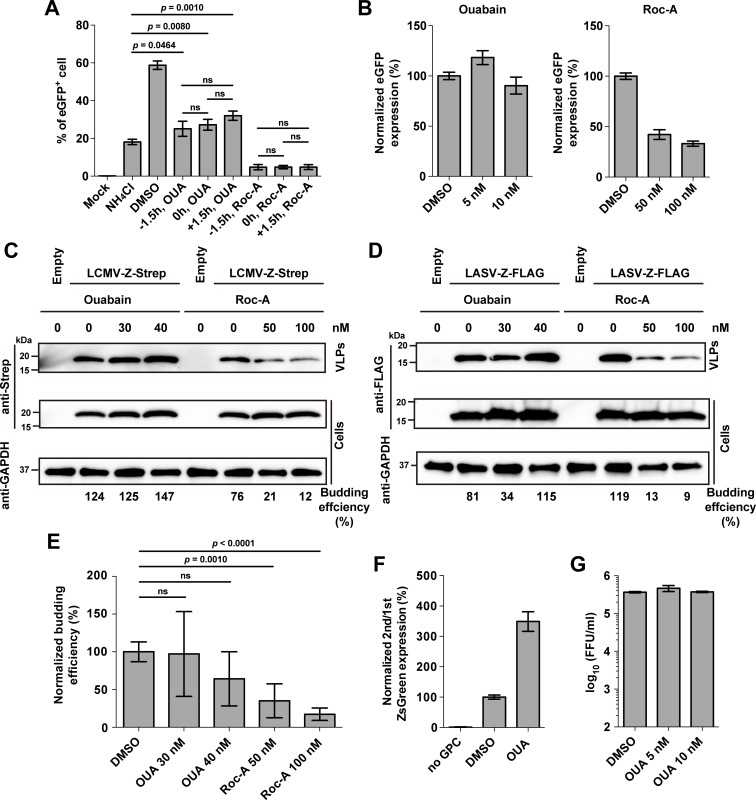
ATP1A1 and PHB are involved in different steps of the mammarenavirus life cycle. **(A)** Influence of time of addition of ouabain (OUA) or rocaglamide (Roc-A) on virus multiplication. A549 cells seeded (2.5 x 10^5^ cells/well) in a 12-well plate and cultured overnight were infected (moi = 0.1) with rLCMV/eGFP or remained uninfected (mock). OUA (10 nM), Roc-A (100 nM), or DMSO (0.01%) was added to the culture media at the indicated time points and remained present throughout the end of the experiment. Ammonium chloride (20 mM) was added to culture medium at 4 h pi to prevent multiple rounds of virus infection. At 24 h pi, eGFP expression in infected cells was examined by flow cytometry. Data represent mean ± SD of the results of three independent experiments. **(B)** Effect of ouabain and Roc-A on LCMV replication. A549 cells seeded (1.25 x 10^5^ cells/well) in 24-well plates and cultured overnight were infected (MOI = 1) with rLCMVΔGPC/eGFP, followed by addition of the indicated concentrations of ouabain or Roc-A. At 72 h pi, total cell lysates were prepared, and eGFP expression levels were measured using a fluorescent plate reader. Data represent mean ± SD of three independent experiments. **(C-E)** Effect of ouabain and Roc-A on Z-mediated budding. Cells (HEK 293T) seeded (3.5 x 10^5^ cells/well) in a 12-well plate and cultured overnight were transfected with 0.5 μg of either pC-Empty or pC-LCMV-Z-Strep (LCMV-Z-Strep) **(C)** or pC-LASV-Z-FLAG (LASV-Z-FLAG) **(D, E)**. At 24 h post-transfection, cells were washed with fresh media to eliminate Z-mediated production of VLPs in the absence of compound treatment, and cultured for another 24 h in fresh media in the presence of ouabain or Roc-A at the indicated concentrations. VLPs present in TCS were collected by ultracentrifugation, and cell lysates were prepared. Z protein expression in VLPs and cell lysates were determined by western blots using antibodies to Strep-tag **(C)** and FLAG-tag **(D)**. Budding efficiency for each sample was estimated by dividing the signal intensity of the Z protein associated with VLPs by that of Z detected in the cell lysate. Numbers on the bottom of panel **C** correspond to LCMV Z budding efficiencies determined in a representative experiment. Results shown in panel **E** correspond to the average and SD from four independent experiments including the one shown in panel **D**. The mean budding efficiency of DMSO treated-samples was set to 100%. Data represent mean ± SD of four independent experiments. **(F)** Effect of ouabain on incorporation of viral glycoprotein into virions. 293T cells seeded (4.0 x 10^5^ cells/well) in a 12-well plate and cultured overnight were infected (MOI = 0.1) with scrLCMV/ZsG (1^st^ infection) for 2 h and subsequently transfected with 0.5 μg of pC-GPC. At 24 h pi, cells were washed with fresh medium to eliminate infectious virus particle produced in the absence of compound treatment, and cultured for another 24 h in fresh media in the presence of ouabain at 40 nM (OUA). At 48 h pi, TCS was collected and used to infect fresh monolayer of BHK-21 cells (2^nd^ infection) seeded (4.0 x 10^5^ cells/well) in a 12-well plate 1 day before the infection, and 293T cell lysate was prepared. 24 h later, BHK-21 cell lysate was prepared. ZsGreen signal intensity was measured by a fluorescent plate reader. GP-incorporation efficiency was estimated by dividing ZsGreen signal intensity in BHK-21 cell lysate (2^nd^) by that in 293T cell lysate (1^st^). The mean GP-incorporation efficiency of DMSO treated samples was set to 100%. Data represent means ± SD from three independent experiments. **(G)** Effect of ouabain on the late stage of LCMV infection. A549 cells seeded (1.25 x 10^5^ cells/well) and cultured overnight were infected (MOI = 0.1) with rLCMV/eGFP. At 48 h pi, cells were washed with fresh medium to eliminate infectious virus particle produced in the absence of compound treatment, and cultured for another 24 h in fresh medium in the presence of ouabain (OUA) at indicated concentrations. At 72 h pi, TCS was collected and virus titers were determined by IFFA. Data represent means ± SD from three independent experiments.

To gain insights about the mechanisms by which ouabain and rocaglamide exert their anti-LCMV activity, we examined effects of these compounds on distinct steps of the LCMV life cycle. First, we asked whether ouabain and rocaglamide affected cell entry of LCMV. We conducted time-of-addition experiments in which we treated cells with ouabain or rocaglamide prior to virus inoculation (-1.5 h), at the time of inoculation (0 h), or 1.5 h pi (+1.5 h) (**Fig 6A**). In some samples, we used ammonium chloride starting at 4 h pi to block multiple rounds of virus infection. The timing of compound addition did not significantly change the number of eGFP-positive cells, indicating that neither ouabain nor rocaglamide inhibited cell entry of LCMV. The number of eGFP^+^ cells in ouabain-treated cells was reduced at all time-of-addition points compared to vehicle dimethyl sulfoxide (DMSO)-treated cells, but was similar to that observed in ammonium chloride-treated cells. Thus, ouabain did not inhibit LCMV RNA replication and gene expression, but rather a late step of the LCMV life cycle. In contrast, rocaglamide treatment resulted in a negligible number of eGFP^+^ cells, indicating that rocaglamide inhibited virus RNA replication and gene transcription.

To further investigate the effect of ouabain and rocaglamide on virus RNA synthesis, we infected A549 cells with a recombinant single-cycle infectious LCMV expressing eGFP (rLCMVΔGPC/eGFP) and treated cells with either ouabain or rocaglamide. Seventy-two hours later, we determined percent normalized eGFP expression in infected cells (**Fig 6B**). Consistent with our results from the time-of-addition experiment, ouabain did not affect reporter eGFP expression. However, rocaglamide reduced eGFP expression, confirming inhibitory effect of rocaglamide on virus RNA synthesis.

We also examined the effect of ouabain and rocaglamide on the late step of the arenavirus life cycle, Z-mediated budding. For this experiment, we transfected cells with Z-Strep- and Z-FLAG (DYKDDDDK epitope)-expressing plasmids from LCMV and LASV, respectively. At 24 h post-transfection, we removed the tissue culture supernatant (TCS) and washed extensively transfected cells to eliminate already budded Z. We cultured the transfected cells in the presence or absence of ouabain or rocaglamide. At 24 h, we determined by WB levels of Z protein in both whole cell lysates and associated with virus-like particles (VLPs) collected from TCS. Treatment with rocaglamide, but not with ouabain, caused a reduction in both LCMV and LASV Z budding efficiency (**Fig 6C and 6D**). The reproducibility of these findings was confirmed based on results from four independent experiments (**Fig 6E**). We also examined whether ouabain could interfere with a step of assembly of infectious progeny that was not captured by the Z budding assay through two additional experiments. The first experiment involved the use of a newly generated single-cycle infectious recombinant LCMV expressing the reporter gene ZsGreen (scrLCMV/ZsG-P2A-NP) to infect (MOI = 0.1) A549 cells (1^st^ infection). These cells were subsequently transfected with a plasmid expressing LCMV GPC. At 24 h pi, we washed the infected cells to remove the extracellular virus produced during the first 24 h of infection, and added fresh media containing ouabain (40 nM) or vehicle control (DMSO).

After 24 h, we used TCS to infect a fresh cell monolayer (2^nd^ infection) and identified infected cells based on ZsGreen expression. To assess the effect of ouabain on de novo assembly of infectious progeny we determined normalized ratios (2^nd^/1^st^ infection) of ZsGreen + cells (**Fig 6F**). The second experiment involved infection (MOI = 0.1) of cells with WT LCMV, and 48 h later we washed infected cells three times to remove the extracellular infectious progeny produced during the first 48 h of infection. Then, fresh media containing ouabain or DMSO vehicle control were added, and 24 h later we determined virus titers in TCS (**Fig 6G**). Results from both experiments consistently showed that ouabain did not inhibit assembly de novo of extracellular infectious virus.

**Fig 7:**
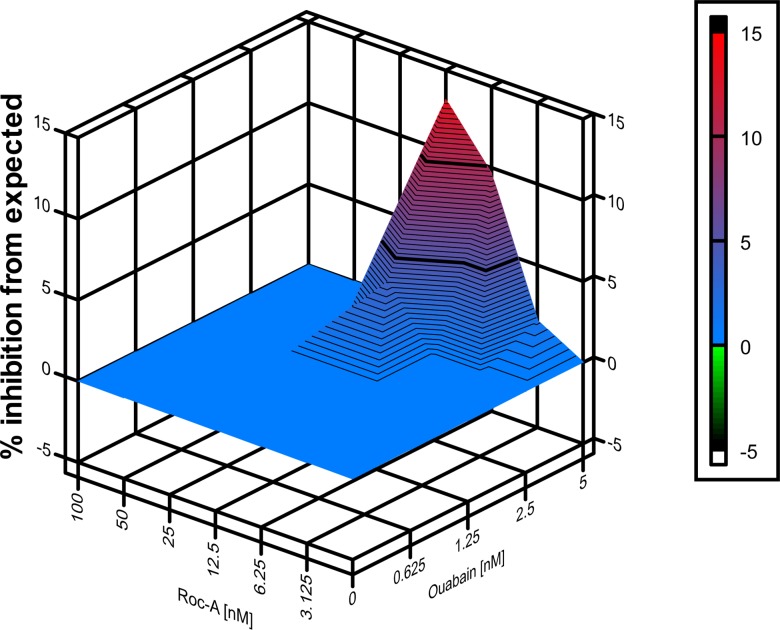
Synergistic antiviral effect of ouabain and rocaglamide on rLCMV/eGFP multiplication. A549 cells seeded (2.0 x 10^4^ cells/well) in 96-well plates and cultured overnight were treated with combinations of ouabain and Roc-A at indicated concentrations for 2 h and then infected (MOI = 0.01) with rLCMV/eGFP. Compounds were present throughout the end of experiment. At 48 h pi, cells were fixed and stained with DAPI. eGFP and DAPI signals were measured by a fluorescent plate reader. eGFP signal was normalized to DAPI signal, and the normalized data were used to analyze synergistic effect by MacSynergy II software. Data represent % synergy (% inhibition over the expected [additive effect]) at the 95% confidence interval from five independent experiments.

Combination therapy can significantly alleviate the problem posed by the rapid emergence of drug-resistant variants commonly observed during monotherapy strategies to control RNA virus infections. Since ouabain and rocaglamide inhibited different steps of the LCMV life cycle, we examined whether their use in combination results in a synergistic anti-LCMV effect. For this experiment, we infected A549 cells with rLCMV/eGFP and treated them with ouabain and rocaglamide using different concentrations and combinations. At 48 h pi, we determined percent eGFP expression (**Fig 7**). Combination treatment with ouabain and rocaglamide resulted in synergistic anti-LCMV activity that was enhanced under conditions using higher concentrations of ouabain and lower concentrations of rocaglamide.

### Effects of pharmacological inhibition of ATP1A1 and PHB on multiplication of LASV and JUNV

**Fig 8:**
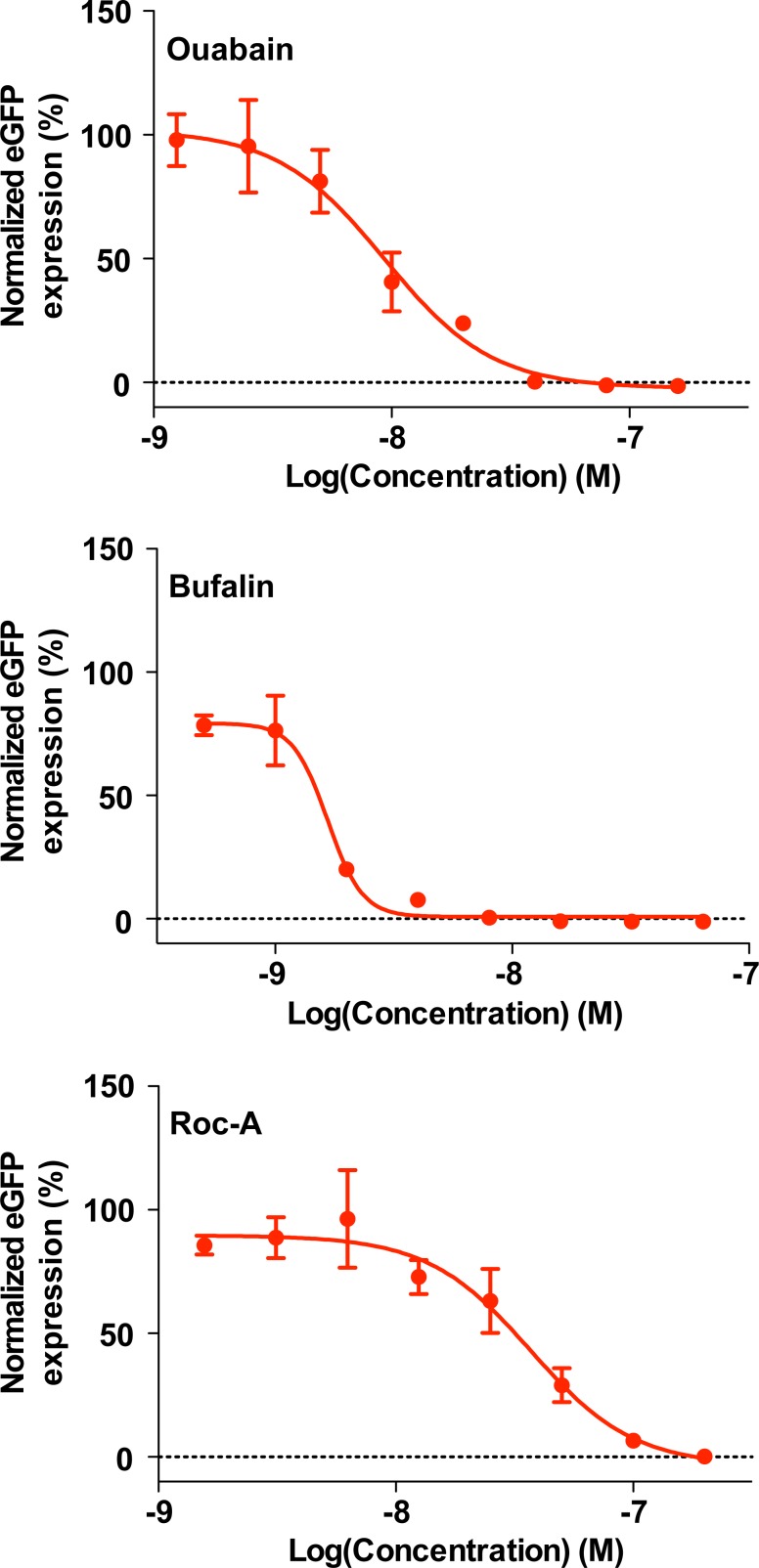
Inhibitory effects of ouabain, bufalin, and rocaglamide on LASV propagation. A549 cells seeded in a 96-well plate (3.0 x 10^4^ cells/well) and cultured overnight were treated with 2-fold serial compound dilutions at 37°C and 5% CO_2_ for 2 h, followed by inoculation with rLASV/eGFP (MOI = 0.01). Compounds were present to study endpoint. At 48 h pi, cells were fixed with 4% PFA in PBS, and eGFP expression was examined by a fluorescent plate reader. Mean values obtained with DMSO-treated and rLASV/eGFP-infected cells were set to 100%. The data represent means ± SD of the results of three replicates.

We next asked whether the ATP1A1 and PHB host-cell factors contributed also to multiplication of viral hemorrhagic fever-causing LASV. We treated A549 cells with ouabain, bufalin, or rocaglamide and inoculated the treated cells with recombinant LASV expressing eGFP (rLASV/eGFP). eGFP expression was examined 48 h later. Similar to rLCMV infection, LASV multiplication was restricted in ouabain-, bufalin-, or rocaglamide-treated cells at concentrations minimally impacting cell viability, although their IC_50_ values were slightly higher than those found with the LCMV infection system (**Fig 5B** and **S2 Fig**) as ouabain had IC_50_ of 9.34 nM, bufalin had IC_50_ of 1.66 nM and rocaglamide had IC_50_ of 37.0 nM (**Fig 8**). We also tested the effect of compounds targeting ATP1A1 and PHB on multiplication of JUNV. Consistent with our results obtained with LCMV and LASV, ouabain, bufalin, and rocaglamide strongly suppressed JUNV multiplication (**S3 Fig**). These findings indicate that ATP1A1 and PHB function as pro-viral factors of a range of mammarenaviruses.

## Discussion

We identified ATP1A1 and PHB as novel host-cell proteins that contribute to efficient multiplication of mammarenaviruses. Our approach using a recombinant LCMV expressing NP with an affinity tag facilitated defining the NP interactome in the context of LCMV infection. Using virus-infected cells, viral protein expression and interactions are regulated by the natural physiological conditions of the infection. These experimental conditions overcome concerns about potential artifactual interactions due to plasmid-mediated overexpression of a single viral protein in transfected cells. Infection with live virus can also overcome the technical complications of transfection efficiency of plasmid-based expression systems, which limits the cell types that can be used to define viral protein interactomes. Our rLCMV/Strep-NP methodology can be used in primary cells, including dendritic cells and monocytes, which are important cell targets during natural mammarenavirus infections but have low transfection efficiency.

Recently, using an NP-specific monoclonal antibody (mAb) to precipitate NP and associated cellular protein partners in a mammarenavirus NP interactome, King *et al*. identified 348 host proteins that associated with LCMV NP [45]. We found 99 common proteins between our filtered LCMV NP interactome of 171 proteins and the LCMV NP interactome documented by King *et al*. Differences in both experimental conditions and analysis methodologies used to generate the LCMV NP interactome documented by King *et al*. and ours likely contributed to the observed differences between data sets. Despite progress in the implementation of global proteomics-based screens to identify virus-host protein-protein interactions, overlap between datasets for the same viral system is usually limited. However, the substantial overlap of 99 of the 171 NP-interacting proteins from both studies supports the overall reliability of both systems. We used results of the eGFP-Strep interactome, determined in r3LCMV/eGFP-Strep-infected cells, as a control to filter out non-specific NP interactions, which may have resulted in a higher degree of stringency than in the study by King *et al* for selection of NP-interacting candidates. The combined information provided by the NP interactome reported by King *et al*. and the one we present in this work, will facilitate future studies to further characterize the functional and biological implications of NP-host cell interacting proteins.

All tested mammarenavirus NPs, with exception of the NP from TCRV, blocked IRF-3-dependent IFN-I induction [25, 46]. The anti-IFN activity of NP was mapped to its C-terminal part and linked to the 3'-5' exonuclease domain present with the NP C-terminal part [30]. Inhibitor-ĸB kinase ε (IKKε) was identified as an NP-binding protein using plasmid-mediated overexpression in transfected cells [47], and the NP-IKKε binding affinity correlated with NP's ability to inhibit IFN-I induction [47]. We, as well as the work by King *et al*. [45], did not detect this NP-IKKε interaction. This discrepancy may have been caused by very low expression of IKKε in LCMV-infected cells, which prevented detection of IKKε by LC-MS/MS. Alternatively, NP-IKKε interaction could possibility be temporarily regulated and take place at early times pi, but could be mostly absent at 48 h pi, the time at which we prepared the cell lysates for our proteomics studies. Future studies comparing the NP interactome at different times during infection will contribute to a better understanding of the dynamics of NP/host-cell protein interactions.

Na^+^/K^+^-ATPase is a well-characterized membrane ion transporter and is composed of two functional subunits (α and β) and one regulatory γ subunit [48]. ATP1A1 represents one of four α subunits [49, 50]. Recent evidence has suggested that the Na^+^/K^+^-ATPase is involved in multiple cell signaling pathways that are independent of its ion-pumping function [51]. Cardiac glycoside inhibitors of the Na^+^/K^+^-ATPase (NKA), so-called cardiotonic steroids (CST; e.g., ouabain, bufalin), have been shown to inhibit multiplication of different viruses including Ebola virus [35], coronaviruses [36], herpes simplex virus 1 [52, 53], CHIKV [54], human immunodeficiency virus 1 (HIV-1) [55], adenovirus [56] and porcine reproductive and respiratory syndrome virus 1 [57].

Different mechanisms are likely to contribute to the antiviral activity of CSTs, including altered cell functions modulated by the signaling activity of Na^+^/K^+^-ATPase [58]. Thus, a low concentration of ouabain induces a conformational change in ATP1A1 that results in activation and release of proto-oncogene tyrosine protein kinase, Src, from ATP1A1, followed by activation of as yet unknown downstream signaling that inhibits, for instance, cell entry of murine hepatitis virus (MHV) [59]. However, our results indicated that ouabain did not interfere with LCMV cell entry. In addition, treatment with the Src inhibitor 4-amino-5-(4-methylphenyl)-7-(t-butyl)pyrazolo[3,4-d]pyrimidine (PP1) did not counteract the anti-LCMV activity of ouabain (**S4 Fig**). Nevertheless, ATP1A1-mediated Src signaling could plausibly contribute to the inhibitory effect of ouabain on JUNV multiplication as similarly to that observed with MHV. Moreover, cell entry of JUNV occurs also by clathrin-mediated endocytosis [60], a process affected by Src signaling. Ouabain has been clinically used in several European countries for the management of congestive heart failure, whereas bufalin has been tested in clinical trials for cancer treatments [61], and the CST digoxin has been FDA-approved since 1997 to treat heart failure and atrial fibrillation. Hence, opportunities for the repurposing CSTs have potential as therapeutics to treat infections caused by viral hemorrhagic fever-causing arenaviruses.

The PHB inhibitor, rocaglamide, appeared to interfere with LCMV RNA synthesis and budding, but did not affect LCMV cell entry. In contrast, PHB was reported to be a cell entry receptor for DENV-2 [39] and CHIKV [40]. On the other hand, PHB did not act as a virus cell entry receptor for HCV. Rather, PHB contributed to HCV cell entry through binding to cellular RAF (c-Raf; proto-oncogene serine/threonine-protein kinase) and subsequent Harvey rat sarcoma proto-oncogene (HRas) activation that induces a signal transduction pathway required for epidermal growth factor receptor (EGFR)-mediated HCV cell entry [37]. In addition, siRNA-mediated kd of PHB decreased production of H5N1 FLUAV [38]. These findings indicate that PHB is involved in different steps of the life cycle of a variety of viruses, and thereby an attractive target for the development of broad-spectrum antiviral drugs.

Rocaglate is a group of natural compounds, which includes rocaglamide, that inhibits protein synthesis by targeting the ATP-dependent DEAD-box RNA helicase eukaryotic initiation factor 4A (eIF4A) and exerts anti-tumor activity [62, 63]. The rocaglate compound, silvestrol, inhibits Ebola virus multiplication likely by interfering with the role of eIF4A in viral protein translation [64].

While we focused on two host proteins, ATP1A1 and PHB, in this study, our proteomics approach also identified several NP-interacting host-cell proteins whose kd expression via siRNA resulted in increased LCMV multiplication. These proteins, which included MAP1B, might have anti-LCMV activity. MAP1B has been shown to bind to nonstructural proteins 1 (NS1) and 2 (NS2) of human respiratory syncytial virus (HRSV) [34]. NS1 and NS2 of HRSV antagonizes host IFN-I response by reducing the levels of TNF receptor associated factor (TRAF3), IKKε (NS1), and STAT2 (NS2) [65]. NS2-MAP1B interaction interfered with HRSV NS2’s ability to reduce levels of STAT2, whereas the role of NS1-MAP1B interaction remains to be determined [34]. Examining the role of NP-MAP1B interaction in modulating NP’s ability to inhibit induction of type I IFN is of interest.

We identified among the NP-interacting host cell proteins the RNA helicase Moloney leukemia virus 10 (MOV10), which has been reported to be an antiviral factor for FLUAV [66], retroviruses [67–71], and DENV-2 [72]. We did not observe increased LCMV multiplication in cells subjected to siRNA-mediated kd of MOV10, a finding that would question an anti-LCMV activity of MOV10. However, we consider that LCMV has already optimal multiplication in A549 cells and further increases may occur only under rather unique conditions. MOV10 was shown to enhance IRF-3-mediated IFN-I induction following SeV infection through a tank binding kinase 1 (TBK1)-independent and IKKε-dependent manner. This finding was further supported by demonstrating MOV10-IKKε interaction by co-immunoprecipitation studies [73]. We documented that the anti-IFN activity of mammarenavirus NP correlated with its ability to interact with IKKε [47]. Whether NP-MOV10 interaction prevents MOV10 from enhancing IRF-3-mediated IFN-I induction remains to be determined.

Several members of the mammalian chaperonin-containing T-complex (CCT) were identified as prominent hits in our NP interactome. The mammalian CCT is critical for folding of many proteins with important functions in diverse cellular processes [74], and may protect complex protein topologies within its central cavity during biosynthesis and folding [75]. The contribution of CCT members to NP assembly into a nucleocapsid structure could account for their presence in the NP, but not eGFP, interactome. Interestingly, members of the CCT have been implicated in multiplication of different viruses including rabies virus [76, 77], HCV [78] and FLUAV [79]. However, the role of these CCT proteins in virus multiplication remains unknown and may involve functions other than acting as molecular chaperones.

Previous studies documented the presence of several components of the of eIF4F, including 4A, within viral replication-transcription complexes (RTC) detected in cells infected with LCMV [80] and TCRV [81]. These findings, together with the detection of a number of ribosomal proteins in the NP interactome, suggest that translation of viral mRNAs may take place within RTC. However, rocaglamide interference with the activity of eIF4A within the viral RTC might contribute to its anti-LCMV activity.

In this work, we documented the generation of rLCMV/Strep-NP and its use to define the NP-interactome in infected cells. We presented evidence that ATP1A1 and PHB contribute to efficient multiplication of mammarenaviruses using genetics and pharmacological inhibition of the genes. Consistent with our findings, bioinformatic analysis revealed that the protein network associated with ATP1A1 and PHB involves host cell proteins with functions in biological processes that have been implicated in virus multiplication (**S5 Fig**). The overall experimental approach described here can facilitate the identification of biologically relevant NP-interacting host-cell proteins. Future studies elucidating the roles of pro- and antiviral host-cell factors identified in this study in mammarenavirus multiplication will advance our understanding of the multiple functions of NP and uncover novel cellular targets for the development of anti-mammarenaviral drugs. In addition, by identifying proviral host-cell factors, drugs that are already approved can be repurposing as therapeutics to combat human pathogenic mammarenavirus infections.

## Materials and methods

### Cells

Baby hamster kidney BHK-21 (American Type Culture Collection, ATCC, CCL-10), house mouse L929 (ATCC CCL-1), grivet Vero E6 (ATCC CRL-1586), human A549 (ATCC CCL-185), and human HEK 293T (ATCC CRL-3216) cells were grown in Dulbecco’s modified Eagle’s medium (Thermo Fisher Scientific, Waltham, MA) containing 10% heat-inactivated fetal bovine serum, 2 mM of L-glutamine, 100 mg/ml of streptomycin, and 100 U/ml of penicillin at 37°C and 5% CO_2_.

### Generation of recombinant viruses

WT recombinant LCMVs, Armstrong (rLCMV ARM) and clone-13 (rLCMV Cl-13) strains, were generated as described [30, 82, 83]. Generation of rLCMV/NP(D382A) and SeV, strain Cantell, was described [30, 82, 83]. An rLCMV lacking GPC and expressing eGFP (rLCMVΔGPC/eGFP) was generated by reverse genetics using procedures previously described [84]. rLCMV/Strep-NP and r3LCMV/eGFP-Strep were generated by reverse genetics using similar procedures to generate WT rLCMV and tri-segmented LCMV (r3LCMV) expressing eGFP [30]. For the generation of these novel rLCMVs, we created pol1S Cl-13 plasmids that directed Pol1-mediated intracellular synthesis of recombinant LCMV S genome RNA species coding for Strep-tagged NP or eGFP, respectively (**Fig 1A and 1B**). The rLCMV expressing eGFP (rLCMV/eGFP) was generated as described [85], and the rLCMV expressing ZsGreen (rLCMV/ZsG) instead of eGFP was generated by reverse genetics using similar procedures to generate rLCMV/eGFP. Generation of rLASV expressing eGFP (rLASV/eGFP) will be described elsewhere. A tri-segmented recombinant live-attenuated Candid #1 strain of JUNV expressing eGFP (r3JUNV/eGFP) was generated as described [86]. For the generation of a novel single cycle rLCMV expressing ZsGreen (scrLCMV/ZsG-P2A-NP), a pol1S plasmid was created by omitting GPC open reading frame (ORF) from pol1S plasmid used for the generation of rLCMV/ZsG. scrLCMV/ZsG-P2A-NP was rescued by reverse genetics using similar procedures to generate rLCMVΔGPC/eGFP [84].

### Virus titration

LCMV titers were determined by immunofocus forming assay (IFFA) as described [87]. Briefly, 10-fold serial virus dilutions were used to infect Vero E6 cell monolayers in a 96-well plate, and at 20 h pi, cells were fixed with 4% paraformaldehyde (PFA) in phosphate-buffered saline (PBS). After cell permeabilization by treatment with dilution buffer (DB) (0.3% Triton X-100 in PBS-containing 3% bovine serum albumin [BSA]), cells were stained with a rat mAb to NP (VL-4, Bio X Cell, West Lebanon, NH) conjugated with Alexa Fluor 488 (VL-4-AF488, Protein Labeling Kit, Life Technologies, Carlsbad, CA). VSV titers were determined by a plaque assay.

### Western blot

Total cell lysates were prepared in PD lysis buffer (+) (250 mM of NaCl, 50 mM of Tris-HCl [pH = 7.5], 0.5% TritonX-100, 10% glycerol, 1 mM of MgCl_2_, 1 μM of CaCl_2_, 1 μM of ZnCl_2_) and clarified by centrifugation at 21,130 x *g* at 4°C for 10 min. Clarified lysates were mixed at a 1:1 ratio with loading buffer (100 mM of Tris [pH 6.8], 20% 2-mercaptoethanol, 4% SDS, 0.2% bromophenol blue, 20% glycerol) and boiled for 5 min. Proteins samples were fractionated by SDS-PAGE using 4–20% gradient polyacrylamide gels (Mini-PROTEAN TGX gels 4–20%, Bio-Rad, Hercules, CA), and proteins were transferred by electroblotting onto polyvinylidene difluoride membranes (Immobilin Transfer Membranes, Millipore, Billerica, MA). To detect Strep-tagged proteins, membranes were reacted with mouse monoclonal antibodies to Strep (QIAGEN, Germantown, MD), eGFP (Takara Bio USA, Mountain View, CA), GP_2_ (We33/36), ATP1A1 (TehrmoFisher Scientific, Rockford, IL), PHB (Abcam, Cambridge, MA) or rabbit polyclonal antibodies to α-tubulin (Cell Signaling Technologies, Danvers, MA) or glyceraldehyde-3-phosphate dehydrogenase (GAPDH, Millipore), respectively, followed by incubation with appropriate horseradish peroxidase-conjugated anti-mouse or anti-rabbit immunoglobulin G (IgG) antibodies (Jackson ImmunoResearch Laboratories, West Grove, PA). SuperSignal West Pico or Femto chemiluminescent substrate (Thermo Fisher Scientific) was used to elicit chemiluminescent signals that were visualized using ImageQuant LAS 4000 Imager (GE Healthcare Bio-Sciences, Pittsburgh, PA).

### Identification of NP-binding host-cell proteins

#### Pull down of strep-tagged proteins from infected cell lysate

A549 cells prepared in six 15-cm dishes (approximately 1.0 x 10^8^ cells in total) were infected with either rLCMV/Strep-NP or r3LCMV/eGFP at an MOI of 0.1. At 48 h pi, cells were washed three times with ice-cold PBS, scraped into fresh ice-cold PBS, and centrifuged at 400 x *g* at 4°C for 10 min. Supernatant was removed, and cells were lysed with 12 ml of PD lysis buffer (+) supplemented with halt protease and phosphatase inhibitor cocktail (Thermo Fisher Scientific) and 5 μg/ml of deoxyribonuclease I (Worthington Biochemical Corporation, Lakewood, NJ). Lysate was clarified by centrifugation at 3,900 x *g* at 4°C for 30 min to remove cell debris. Clarified cell lysate was then incubated with strep-tactin sepharose resin (QIAGEN) at 4°C. After 2 h of incubation, the resin was washed three times with PD lysis buffer (+) and once with PD lysis buffer without TritonX-100 (PD lysis buffer [–]). After the centrifugation at 1,600 x *g* and 4°C for 5 min, the last wash buffer was removed, and protein complexes associated with the resin were eluted into 2 ml of PD lysis buffer (-) containing 2.5 mM of desthiobiotin. The eluate was then subjected to TCA precipitation followed by trypsin digestion.

#### Multidimensional protein identification technology microcolumn

A MudPIT microcolumn was prepared by first creating a Kasil frit at one end of an un-deactivated 250-μm outside diameter (OD) capillary tube (interior diameter of 360 μm)(Agilent Technologies, Inc., Santa Clara, CA). The Kasil frit was prepared by briefly dipping a 20–30-cm capillary tube in 300 μl of Kasil 1624 potassium silicate well-mixed solution (PQ Corporation, Malvern, PA) and 100 μl of formamide, curing at 100°C for 4 h, and cutting the frit to a length of ≈2 mm. Strong cation exchange particles (SCX Luna, 5-μm diameter, 125 Å pores, Phenomenex, Torrance, CA) were packed in-house from particle slurries in methanol to 2.5 cm. Reversed phase particles (2 cm, C18 Aqua, 3-μm diameter, 125 Å pores, Phenomenex) were then successively packed onto the capillary tube using the same method as SCX loading.

#### MudPIT analysis

An analytical reversed-phase liquid chromatography column was generated by pulling a 100-μm (interior diameter (ID) of 360 μm) OD capillary tube (Polymicro Technologies, Phoenix, AZ) to 5-μm ID tip. Reversed-phase particles (Luna C18, 3-μm diameter, 125 Å pores, Phenomenex) were packed directly into the pulled column at 5.5 mPa until 15 cm long. The column was further packed, washed, and equilibrated at 10 mPa with buffer B (80% acetonitrile, 0.1% formic acid) followed by buffer A (5% acetonitrile and 0.1% formic acid). MudPIT and analytical columns were assembled using a zero-dead volume union (Upchurch Scientific, Oak Harbor, WA). LC-MS/MS analysis was performed with an Agilent high-pressure LC pump (Agilent) and linear quadrupole ion dual cell trap Orbitrap Velos (Thermo) using an in-house built electrospray stage. Electrospray was performed directly from the analytical column by applying the electrospray ionization (ESI) voltage at a tee (150 μm ID, Upchurch Scientific) directly downstream of a 1:1,000 split flow to reduce the flow rate to 300 nl/min through the columns. MudPIT experiments (10-step) were performed in which each step corresponds to 0, 10, 20, 40, 50, 60, 70, 80, 90, and 100% buffer C (500 mM of ammonium acetate, 0.1% formic acid, and 5% acetonitrile) and was run for 3 min at the beginning of a 110-min gradient.

#### Data analysis

Protein and peptide identification were performed with Integrated Proteomics Pipeline—IP2 (Integrated Proteomics Applications, San Diego, CA. http://www.integratedproteomics.com/) using ProLuCID and DTASelect2 algorithms. DTASelect parameters were—p 2 -y 1—trypstat—pfp .01 –extra-pI-DB-dm-in. Spectrum raw files were extracted into ms2 files from raw files using open source RawExtract 1.9.9 (Scripps Research Institute, La Jolla, CA; http://fields.scripps.edu/downloads.php), and the tandem mass spectra were searched against a human protein database (UniprotKB). To accurately estimate peptide probabilities and false discovery rates, we used a decoy database containing the reversed sequences of all the proteins appended to the target database. Tandem mass spectra were matched to sequences using the ProLuCID algorithm with a 600-ppm peptide mass tolerance. ProLuCID searches were done on an Intel Xeon cluster processor running under the Linux operating system. The search space included half and fully tryptic peptide candidates that fell within the mass tolerance window with no miscleavage constraint. Carbamidomethylation (+57.02146 Da) of cysteine was considered as a static modification.

### siRNA screening

A549 cells (1,000 cells/well) in a 384-well plate were reverse transfected with 0.5 pmol of siRNA pool (**S2 Table**) targeting each gene using 0.1 μl of Lipofectamine RNAiMAX (Thermo Fisher Scientific) (final siRNA concentration was 10 nM), followed by incubation at 37°C and 5% CO_2_. At 72 h post-transfection, cells were infected (MOI = 0.05) with rLCMV/ZsG. siRNA target host-cell proteins were selected based on availability of validated siRNA sequences. The siRNAs we used to examine the effects on LCMV multiplication of knockdown expression of NP-interacting host cell protein candidate hits corresponded to the Genome-wide ON TARGET-Plus (OTP) Human siRNA library (18,301 genes, 4 siRNAs/gene; Dharmacon, Lafayette, CO).

### Verification of siRNA knockdown of genes

A549 cells (3.0 x 10^4^ cells/well) were reverse transfected in a 24-well plate with 6 pmol of siRNA pools targeting each gene using 1 μl of Lipofectamine RNAiMAX (final siRNA concentration is 10 nM). At 72 h post-transfection, total cell lysate was prepared in modified lysis A buffer (25 mM Tris-HCl [pH = 8.0], 50 mM NaCl, 1%Triton X-100, 1.25% sodium deoxycholate) and clarified by centrifugation at 21,130 x *g* at 4°C for 10 min. The total protein concentration of clarified cell lysate was measured by Pierce BCA Protein Assay Kit (Thermo Fisher Scientific). The same amount of protein from each sample was subjected to SDS-PAGE, and the protein expression of siRNA-targeted genes was analyzed by western blots.

### Measurement of green fluorescence and DAPI signals

Cells infected with eGFP- or ZsGreen-expressing rLCMV were fixed with 4% PFA in PBS. After cell permeabilization by treatment with DB, cells were stained with 4',6-diamidino-2-phenylindole (DAPI). Green fluorescence (eGFP or ZsGreen) and DAPI signals were measured by a fluorescent plate reader (Synergy H4 Hybrid Multi-Mode Microplate Reader, BioTek, Winooski, VT).

### Immunofluorescence assay and co-localization analysis

Mock- and virus-infected cells were fixed with 4% PFA. After cell permeabilization and blocking by treatment with DB containing 1% normal goat serum, cells were incubated with primary mouse anti ATP1A1 or PHB antibody followed by secondary anti-mouse IgG antibody conjugated with Alexa Fluore 568 (anti-mouse IgG-AF568). Subsequently, cells were stained with VL-4-AF488. In some samples, primary antibody against ATP1A1 or PHB was omitted to determine background fluorescence. To visualize nuclei, DAPI Fluoromount-G (SouthernBiotech, Birmingham, AL) was used to mount coverslips on a slide glass. Stained cells were observed under a confocal microscope (LSM 710, Zeiss) and data analyzed by ZEN software (Zeiss). Co-localization analysis was performed on a pixel by pixel basis using Zen software (Zeiss). Eight green (NP- positive) cells were marked and every pixel in the marked area was plotted in the scatter diagram based on its intensity level from each channel. Thresholds for green and red channels were determined using mock-infected cells stained with VL-4-AF488 (anti-NP) and anti-mouse IgG antibody conjugated with Alexa Fluor 568, without using anti-ATP1A1 or -PHB antibodies. Each pixel was assigned a value of 1. Co-localization coefficients (CC) (or non-weighted CC) were determined by dividing the sum of both green-and red-positive pixels by the sum of green positive pixels. This calculation was repeated for eight individual cells. To assess the specificity of co-localization, we determined weighted CC by taking into consideration the brightness of each channel signal. Comparison of non-weighted and weighted CC allowed us to determine whether brighter pixels were present in the co-localized regions compared to the non-co-localized regions. *p* values were determined by a two-tailed paired *t* test using GraphPad Prism software.

### IC_50_ determination

A549 or Vero E6 cells seeded (2.0 x 10^4^ cells/well) in a 96-well plate and cultured overnight were treated with 3-fold serial compound dilutions at 37°C and 5% CO_2_ for 2 h, followed by infection with rLCMV/eGFP (MOI = 0.01). Compounds were present to study endpoint. At 48 h pi, cells were fixed with 4% PFA in PBS, and eGFP expression was examined by a fluorescent plate reader (Synergy H4 Hybrid Multi-Mode Microplate Reader, BioTek). Mean values obtained with DMSO-treated and rLCMV/eGFP-infected cells were set to 100%. The IC_50_ concentrations were determined using GraphPad Prism.

### CC_50_ determination

A549 or Vero E6 cells seeded in a 96-well plate (2.0 x 10^4^ cells/well) and cultured overnight were treated with 3-fold serial compound dilutions and cultured at 37°C and 5% CO_2_ for 48 h. Then, CellTiter 96 AQ_ueous_ one solution reagent (Promega, Madison, WI) was added. Thereafter, the assay was performed according to the manufacturer’s recommendations, and the absorbance (490 nm) was obtained using an enzyme-linked immunosorbent assay (ELISA) reader (SPECTRA max plus 384; Molecular Devices, Sunnyvale, CA). Mean values obtained with DMSO-treated cells were set to 100%. The CC_50_ concentrations were determined using GraphPad Prism.

### Analysis of virus replication and gene expression

Cells were infected (MOI = 1) with rLCMVΔGPC/eGFP for 1.5 h or remained uninfected (mock). Compounds were then added to TCS. At 72 h pi, total cell lysates were prepared in cell lysis buffer (150 mM of NaCl, 50 mM of Tris-HCl [pH = 7.5], 0.5% nonyl phenoxypolyethoxylethanol [NP-40], 1 mM of ethylenediaminetetraacetic acid [EDTA]) and clarified by centrifugation at 21,130 x *g* at 4°C for 10 min. The total protein concentration of clarified cell lysate was measured by Pierce BCA Protein Assay Kit (Thermo Fisher Scientific). eGFP levels in clarified cell lysates adjusted to same total protein concentration with cell lysis buffer were measured by a fluorescent plate reader (Synergy H4 Hybrid Multi-Mode Microplate Reader, BioTek).

### Budding assay

A plasmid expressing C-terminus Strep-tagged Z protein (pC-LCMV-Z-Strep) was generated using similar procedure to generate a plasmid expressing C-terminus FLAG-tagged LASV Z protein (pC-LASV-Z-FLAG), and the budding assay was performed as previously described [88]. Cells (HEK 293T) in a 12-well plate were transfected with 0.5 μg of empty pCAGGS vector or pC-LCMV-Z-Strep or pC-LASV-Z-FLAG using Lipofectamine 2000. At 5 h post-transfection, media were replaced with fresh media and incubated at 37°C and 5% CO_2_ for 19 h. Then the cells were three times washed with fresh medium. After the removal of the last wash medium, cells were cultured in fresh medium containing ouabain (30 or 40 nM) or rocaglamide (50 or 100 nM) or equivalent concentration of DMSO, and 24 h later, virion-like particle (VLP)-containing TCS and cells were collected. Total cell lysate was prepared by lysing the cells with lysis buffer (1% NP-40, 50 mM of Tris-HCl [pH 8.0], 62.5 mM NaCl, 0.4% sodium deoxycholate). After clarification of TCS from cell debris by centrifugation at 400 x *g* and 4°C for 10 min, VLPs were collected by ultracentrifugation at 100,000 x *g* and 4°C for 30 min through a 20% sucrose cushion. VLPs were resuspended in PBS, and Z expression in total cell lysate and TCS (containing VLPs) were analyzed by western blots.

### Flow cytometry

A549 cells infected with rLCMV/eGFP were harvested using Accutase cell detachment solution (Innovative Cell Technologies, San Diego, CA) and fixed with 4% PFA in PBS. eGFP expression was examined by flow cytometry using a BD LSR II (Becton Dickson), and data were analyzed with FlowJo (Tree Star, Inc., Ashland, OR).

### Incorporation of viral glycoprotein into mature infectious viral particles

293T cells seeded (4.0 x 10^5^ cells/well) in a 12-well plate and cultured overnight were infected with scrLCMV/ZsG-P2A-NP for 2 h and subsequently transfected with 0.5 μg of pC-GPC. At 24 h pi, cells were three times washed with fresh media to eliminate infectious virus particle produced in the absence of compound treatment, and cultured for another 24 h in fresh media in the presence of 40 nM of ouabain or vehicle control (DMSO). At 48 h pi, TCS was collected and used to infect fresh monolayers of BHK-21 cells seeded (4.0 x 10^5^ cells/well) in a 12-well plate 1 day before the infection, and 293T cell lysate was prepared. 24 h later, BHK-21 cell lysate was prepared. Total cell lysate was prepared in cell lysis buffer (150 mM of NaCl, 50 mM of Tris-HCl [pH = 7.5], 0.5% [NP-40], 1 mM of EDTA] and clarified by centrifugation at 21,130 x *g* at 4°C for 10 min. ZsGreen signal intensity in clarified cell lysate was measured by a fluorescent plate reader (Synergy H4 Hybrid Multi-Mode Microplate Reader, BioTek).

### Assessment of synergy in combination compounds treatment

A549 cells seeded (2 x 10^5^ cells/well) in a 96-well plate and cultured overnight were treated with combinations of different concentrations of ouabain and rocaglamide for 2 h and then infected (MOI = 0.01) with rLCMV/eGFP. Compounds were present in the culture medium throughout the experiment. At 48 h pi, cells were fixed, permeabilized by treatment with DB, and stained with DAPI. eGFP and DAPI signals were measured by a fluorescent plate reader (Synergy H4 Hybrid Multi-Mode Microplate Reader, BioTek). eGFP readouts were normalized by DAPI readouts, and normalized data were used to analyze synergistic effect of the two compounds by the MacSynergy II program [89].

### Statistics

Data were analyzed for *p* values by a two-tailed unpaired *t* test using GraphPad Prism software.

## Supporting information

S1 Fig Effect of pharmacological inhibition of ATP1A1 and PHB on LCMV multiplicaiton. **(A, B)** Effects of ouabain and rocaglamide (Roc-A) on LCMV propagation. A549 (1.25 x 10^5^ cells/well), HEK 293T (1.75 x 10^5^ cells/well), Vero E6 (1.25 x 10^5^ cells/well), L929 (1.25 x 10^5^ cells/well), or BHK-21 (1.75 x 10^5^ cells/well) cells seeded in 24-well plates and cultured overnight were pretreated with either ouabain **(A)** or Roc-A **(B)** at the indicated concentrations or with equivalent concentrations of DMSO for 2 h, followed by inoculation with rLCMV/eGFP at an MOI of 0.01 (A549, 293T, Vero E6, and BHK-21 cells) or 0.1 (L929 cells). At 48 h pi, eGFP expression was observed by a fluorescent microscope.
(EPS)

S2 Fig Effect of bufalin on LCMV propagation and cell viability. A549 and Vero E6 cells seeded in a 96-well plate (2.0 x 10^4^ cells/well) and cultured overnight were treated with 3-fold dilutions of bufalin for 2 h and then infected (moi = 0.01) with rLCMV/eGFP. Bufalin was present throughout the end of experiment. At 48 h pi, cells were fixed to examine eGFP expression and cell viability as determined by CellTiter 96 AQ_ueous_ one solution reagent. The data represent means ± SD of the results of four (cell viability assay) or six (virus spread assay) replicates.
(EPS)

S3 Fig Effect of chemical inhibition of ATP1A1 and PHB on JUNV multiplication. **(A, B)** Effects of ouabain, bufalin, and rocaglamide (Roc-A) on JUNV multiplication. Vero E6 cells seeded in a 24-well plate (1.25 x 10^5^ cells/well) and cultured overnight were inoculated (moi = 0.01) with r3JUNV/eGFP followed by addition of indicated concentrations of ouabain, bufalin, or Roc-A, or equivalent concentration of DMSO to TCS. At 24 h **(A)** and 48 h **(B)** pi, eGFP expression in infected cells was examined by a fluorescent microscope.
(EPS)

S4 Fig Src signaling is not involved in inhibitory effect of ouabain on LCMV multiplication. A549 cells seeded (2.0 x 10^4^ cells/well) in 96-well plates and cultured overnight were treated with 3-fold serial dilutions of a Src inhibitor, PP1, in the presence or absence of 10 nM of ouabain for 2 h and then inoculated (MOI = 0.01) with rLCMV/eGFP. Compounds were present throughout the experiment. At 48 h pi, cells were fixed and stained with DAPI. eGFP and DAPI signals were measured by a fluorescent plate reader. eGFP signal was normalized to DAPI signal. The mean normalized value of vehicle (DMSO)-treated to rLCMV/eGFP-infected sample was set to 100%. Data represent means ± SD of the results from six replicates.
(EPS)

S5 Fig Network analysis associated with ATP1A1 and PHB. Bioinformatic analysis by GeneMANIA was performed showing protein network associated with ATP1A1 and PHB **(A)** and list of functions where those proteins are involved **(B)**.
(EPS)

S1 Table Complete list of host-cell proteins identified in pull down samples with spectral count of 2 or higher. (XLSX)

S2 Table siRNA pool sequences used in Fig 3A. (XLSX)
