# Correction: Interactome analysis of the lymphocytic choriomeningitis virus nucleoprotein in infected cells reveals ATPase Na^+^/K^+^ transporting subunit Alpha 1 and prohibitin as host-cell factors involved in the life cycle of mammarenaviruses

**DOI:** 10.1371/journal.ppat.1008289

**Published:** 2020-01-06

**Authors:** Masaharu Iwasaki, Petra Minder, Yíngyún Caì, Jens H. Kuhn, John R. Yates, Bruce E. Torbett, Juan C. de la Torre

The following information is missing from the Funding statement: This research was supported by the US National Institute of General Medical Sciences (NIGMS) (https://www.nigms.nih.gov/) grant U54 GM103368 to BET, and the US National Institute of Allergy and Infectious Diseases (NIAID) (https://www.niaid.nih.gov/) grant U54 AI150472 to BET.
